# Spatial biases in visual feature representation of mouse dorsal lateral geniculate nucleus boutons

**DOI:** 10.1016/j.isci.2026.117088

**Published:** 2026-08-08

**Authors:** Kuwook Cha, Aline Giselle Rangel Olguin, Reza Sharif-Naeini, Erik P. Cook, Arjun Krishnaswamy

**Affiliations:** 1 Department of Physiology, McGill University, Montreal, QC, Canada

**Keywords:** LGN, visual field specialization, early visual system, neural circuitry, two-photon imaging, axon-localized calcium indicators, chemogenetics, geniculocortical interactions, thalamocortical feedback

## Abstract

Humans and other mammals show spatial biases in their perception of visual features. Although the primary visual cortex is considered a source of these biases, earlier structures may also contribute. Recent work shows feature biases in the retina, but little is known about how they evolve as signals flow through the dorsal lateral geniculate nucleus of the thalamus (dLGN). Using *in vivo* calcium imaging, we investigated spatial frequency, orientation, direction, and temporal frequency representations in both the retina and dLGN boutons. We found modest location-dependent biases in the representation of each feature across visual space for boutons, while such biases were weaker in the retina. dLGN representations emerged from functionally and anatomically defined bouton subsets. Selective ablation of cortical feedback to dLGN modulated feature biases but did not eliminate them. Together, these results suggest that dLGN integrates retinal and cortical inputs to create spatially biased feature maps for V1.

## Highlights

• dLGN boutons display modest location-dependent biases for visual features
• Location-dependent biases for the same visual features are weaker in the retina
• Cortical feedback modulates dLGN location-dependent feature biases

## Graphical abstract

**Figure:**
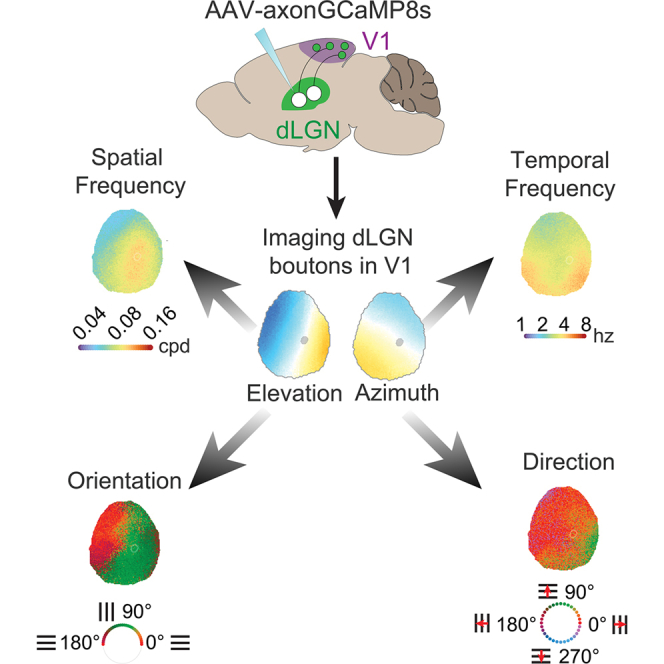

## Teaser

Biological sciences

## Introduction

A growing number of studies suggest the mouse early visual system contains both spatially uniform and non-uniform representations of incoming visual features.^1^^,^^2^^,^^3^ These representations are believed to have evolved to effectively capture both invariant visual features (appearing anywhere in the scene) and those showing spatial biases due to the environment (e.g., color/luminance separation at the horizon) or due to behavior (e.g., radial movement of textures during locomotion).^1^^,^^2^^,^^3^

Classical work supports a role for the geniculocortical pathway in spatially invariant representations, whereas newer work points to the retinotectal pathway in spatially biased ones. For example, representations in the mouse tectum, or superior colliculus (SC), are biased toward certain stimulus orientations and movement directions depending on visual location.^4^^,^^5^^,^^6^^,^^7^^,^^8^^,^^9^ However, similar biases are also seen in the visual cortex (V1) of several species,^10^^,^^11^^,^^12^^,^^13^^,^^14^^,^^15^^,^^16^ including humans, and are thought to underlie perceptual biases for orientation and motion.^15^^,^^17^ Moreover, work in mouse retina also shows spatial biases in feature representation.^3^^,^^18^^,^^19^^,^^20^^,^^21^^,^^22^^,^^23^^,^^24^^,^^25^ These observations suggest that biased and unbiased feature representations may be intermingled across cortically and tectally directed early visual pathways; however, a major node in this system, the dorsal lateral geniculate nucleus (dLGN), remains largely unstudied in this regard.

The dLGN is the sole direct link between the retina and V1,^26^^,^^27^ and its neurons receive input from retinal ganglion cells (RGCs)—retinal neurons comprising ∼50 different types that encode unique features such as motion or edges.^28^^,^^29^^,^^30^^,^^31^ The way RGC types innervate dLGN neurons is still under investigation, but recent work suggests that dLGN neurons may enhance or recombine these incoming retinal feature signals. For example, different direction-selective RGC types tend not to innervate the same dLGN neuron.^32^^,^^33^ *In vivo* recordings show direction- and orientation-tuned dLGN neurons^34^^,^^35^^,^^36^^,^^37^ and suggest that dLGN visual responses resemble that of a single RGC type or are a weighted sum of multiple RGC types.^38^^,^^39^ This matches single-cell rabies tracing showing dLGN neurons connected to a single RGC type or several RGC types.^40^ Thus, subsets of dLGN neurons are feature-selective, but does this selectivity or that of their inputs to V1 vary based on visual location?

To address this issue, we delivered axon-localized calcium indicators^41^ (axon-GCaMP8s) to the dLGN and imaged their nerve terminals across the surface and depth of V1. Using widefield imaging, we observed that dLGN boutons exhibit gradients of preferred spatial frequency, orientation, direction, and temporal frequency. Two-photon imaging showed that these feature gradients were driven by feature preferences of individual boutons, which varied systematically with retinotopic position. But the same experiment detected weak or absent feature gradients across the retinal surface. Finally, genetic ablation of V1-dLGN feedback produced selective deficits in each bouton feature gradient but did not disrupt them entirely. Taken together, our results suggest that dLGN circuits bias the representation of visual features based on retinotopic position.

## Results

### Spatial organization of visual feature preference in mouse dLGN afferents

**Figure 1:**
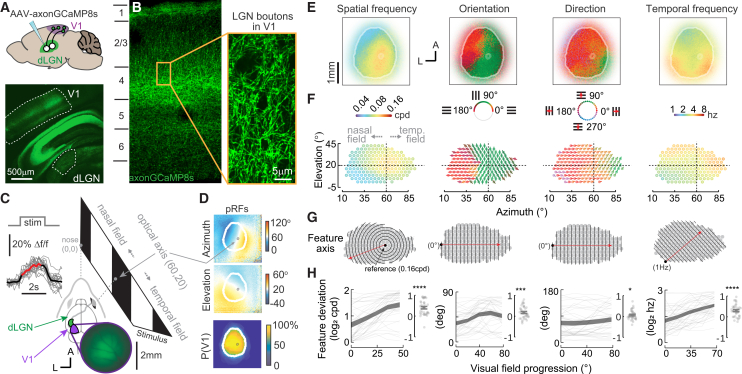
Spatially organized preference for visual features in widefield imaging of dLGN inputs to V1 (A) *Top:* cartoon depicts viral injection of axon-localized GCaMP8s into dLGN to label boutons innervating V1. *Bottom:* confocal image of GCaMP8s expression in a coronal brain slice. dLGN and V1 indicated. Scale bars, 500 μm. (B) Cross section through V1 showing axon-localized GCaMP8s+ dLGN axons. Inset shows a magnified view. Scale bars, 5 μm. (C) Cartoon depicts a head-fixed mouse viewing stimuli while axon-GCaMP8s fluorescence is collected from a cranial window over V1, with anterior (A) and lateral (L) indicated. Example of single-trial (gray) and average responses to a drifting grating. Red is the part of the trace used for further analysis. (D) Standardized azimuth and elevation population receptive field positions (pRFs) and probability map of V1 (P(V1)). White contours delineate the boundary of V1 defined by retinotopy in at least half of the subjects (P(V1) ≥ 0.5). The maps of individual mice were aligned at the region corresponding to the optical axis of the eye, which was the screen center (small gray blob). (E) Average preferred feature maps of dLGN boutons across V1. (F) Maps from E transformed into 2.5^0^ hexagonal bins across visual space. The dashed lines intersect at the optical axis (60, 20). (G) Axes (red arrow) over which feature preference varies in F. Black dot is the reference feature from which the feature deviation in H computed. (H) Deviation of feature preference with receptive field progression along axes in G. Thin lines are individuals and thick lines are mean ± SEM. Inset shows normalized slopes (gray dots) computed from each mouse and the mean ± SEM (*n* = 36 mice, one-sided *t* test; ∗∗∗∗*p* < 10^−5^, ∗∗∗*p* < 10^−3^, and ∗*p* < 0.05).

While retinal inputs are the main drivers of dLGN activity, inputs from V1 also play an important role in modulating dLGN signals.^26^ To preserve this V1 input and measure dLGN feature representation across retinotopic space, we injected mouse dLGN with adeno-associated viruses (AAVs) bearing axon-GCaMP8s^41^^,^^42^ using an angled track that avoided overlying cortex (Figures 1A and 1B).

Tissue sections taken through dLGN following expression showed enriched somatic labeling (via co-expressed Ruby) as compared to the lateral posterior nucleus (LP), whose neurons are also known to innervate V1 as well as higher visual areas (Figures S1A and S1B). Cortical sections from the same mice showed classical features of dLGN innervation of V1^41^^,^^43^^,^^44^^,^^45^ with enriched layer 4 labeling tapering into layers 2/3 and 1 as well as sparse fibers in layer 6 (Figures S1C and S1D). On the other hand, injections in the LP labeled layers 1 and 5 with sparse signals in layer 6, consistent with prior reports^44^^,^^45^^,^^46^ (Figures S1C and S1D). These results indicate that our injection strategy labels dLGN projections to V1.

Following expression, we implanted cranial windows situated over V1 and widefield imaged GCaMP8s signals from dLGN bouton populations while mice viewed visual stimuli (Figures 1C and S1E). We began with a long bar that moved horizontally or vertically to map retinotopic organization (Figures S1E and S1F). Conventional phase and field sign mapping methods^47^ associated dLGN bouton activity with bar position across V1 (Figure S1F). A small (4-8^0^) spot flashed at the screen center (Figures 1C and 1D, *small gray blob)* let us align and standardize cortical space across subjects and obtain population receptive field positions for each pixel (Figure 1D, *see* STAR Methods).

We next presented sets of static gratings, drifting gratings, and full-field luminance-oscillations to measure spatial frequency, orientation, direction, and temporal frequency preferences in the dLGN boutons across V1 (Figure S1E); similar stimuli have been used to define feature selectivity of RGC types.^48^ Bouton population representations for spatial frequency, orientation, direction and temporal frequency were not uniform across V1 (Figure 1E), suggesting feature preference differs across the visual field. To reveal this organization in visual space, we transformed bouton population feature representations from cortical to visual space using pixelwise receptive field positions (Figure 1F, *see* STAR Methods).

These feature maps showed that dLGN bouton populations representing the optical axis of the eye preferred higher spatial frequencies than those representing more eccentric locations (Figure 1F, *spatial frequency*). Bouton populations responsive to the nasal visual field were biased to horizontal orientations, whereas those viewing temporal locations preferred vertical ones with little change in preference across elevation (Figure 1F, *orientation*). Posterior motion was preferred at most azimuths except in the temporal visual field where upward motion was preferred (Figure 1F, *direction*). Finally, dLGN bouton populations representing higher elevations in the temporal visual field were tuned to higher temporal frequencies than those representing lower elevations in the nasal field (Figure 1F, *temporal frequency*). In addition to preference, we also examined feature selectivity and observed that it was uniformly high across the visual field (selectivity index >0.2 for all 4 feature domains, Figure S2A).

Although the modest spatial location biases were evident in population average maps (Figures 1F and S3A), maps from individual mice were more variable (Figure S3B); intersubject variability was particularly large for direction and temporal frequency maps. To determine if the average feature organization was shared across individuals, we quantified it by expressing the relative change in feature preference with progression along characteristic axes in the visual field (Figures 1G and S2B–S2E). Briefly, we computed the deviation of the feature values of all bins (across the entire map) from a reference feature for each map (Figure 1G). This allowed us to express the relative change in feature preference with progression along characteristic axes in the visual field (*visual field progression*, Figures S2B–S2E).

As indicated by the slope of the linear fit, feature preferences deviated significantly with visual field progression (away from the reference) for all four feature maps (Figure 1H). These maps did not result from fisheye distortion and did not shift when we rotated the screen, indicating that our gradients result from biases in the feature preference of dLGN bouton populations rather than being an artifact of the projection of a flat stimulus onto a spherical eye (Figures S4A–S4E). We also did not see these patterns in non-fluorescent control mice, indicating that our results do not arise from intrinsic flavoprotein signals (Figures S4F and S4G). Moreover, our gradients were produced by full-field stimuli and appeared within the screen center, unlike recent reports showing that orientation gradients can form where stimuli interact with a stimulus aperture^49^ or screen edge.^50^ Thus, the widefield results show that the feature preferences of dLGN bouton populations vary across modest gradients within the visual field.

### Feature organization at single bouton-level resolution

The gradients in our average maps indicate that spatially biased feature representations converge across the population of mice. However, variability across mice (Figure S3B) and within mice (see Figure S12) made these gradients appear more modest than those seen in the SC. Note that we include a table that summarizes the statistical strength and effect size of all our results (Table S1).

Variability could come from several sources: (1) inherent representational variability,^8^^,^^51^^,^^52^ (2) variability in expression of axonGCaMP8s, (3) variability introduced by hemodynamic processes that interfere with axonGCaMP fluorescence,^53^ or (4) variability due to differing bouton populations carrying spatially biased and spatially invariant feature representations. We investigated these possibilities by two-photon (2p) imaging of 67 fields from 11 mice across retinotopic positions and depths (for example, fields and Suite2p ROIs see Figure S5). Responsive boutons for each stimulus set were selected (73% of 519,603 boutons detected by Suite2p)^54^ and only a small set of boutons (16%) were responsive to all 3 stimulus sets, indicating bouton responsiveness is stimulus-specific (Figure S6A). Most of the responsive boutons (95%) had their RF within 30^0^ of the field’s population RF location, and the remaining boutons (5%) were excluded (*see* STAR Methods*,* Figure S6B).

**Figure 2:**
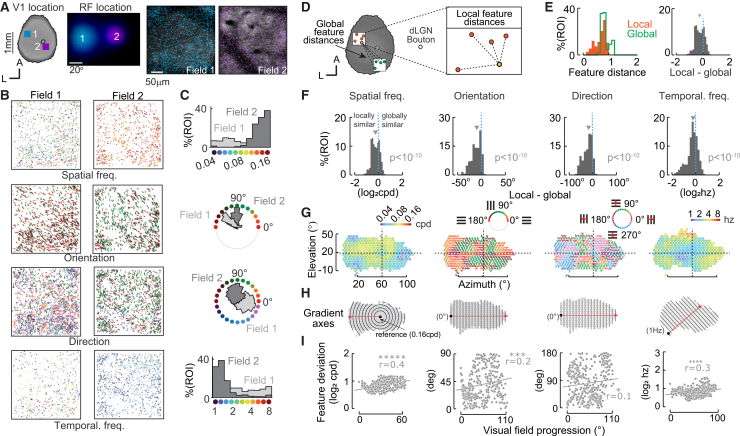
Two-photon imaging of bouton feature preferences reveals local similarity and global organization (A) Cartoon of example two-photon fields in layer 2/3 of V1 (*left*, small gray blob is optical axis) and their corresponding population receptive fields (*middle*), and average images with responsive bouton ROIs indicated (right). Anterior (A) and lateral (L) indicated. Scales: 1 mm (left), 20 deg (middle), 50 μm (right). (B and C) Maps (B) and histograms (C) of preferred spatial frequency, orientation, direction, and temporal frequency preference for the fields in A. Responsive boutons are stimulus-specific. (D) Cartoon shows global and local feature distance metrics for a single bouton. Median feature distance between every bouton (yellow dot) and its within-field members (local, orange) was compared to the median distance to boutons in all other fields (global, green dots). Anterior (A) and lateral (L) indicated. (E) Histogram of local and global feature distances (left) and their difference (right) for an example layer 2/3 field of boutons. (F) Histograms of local minus global feature differences for spatial frequency, orientation, direction, and temporal frequency. Distributions are negatively skewed (*p* < 10^−10^ in all 4 feature domains, sign-test, *n* = 76,707 [SF/Ori], 96,108 [Dir], 66,673 [TF] boutons). All responsive boutons used regardless of layer. (G–H) Bouton feature preferences binned across the visual field from all layers (G) and gradient axes used to measure organization (H). Dashed lines intersect at optical axis (60, 20). (I) Feature deviation versus visual field progression computed from G using axes in H. Lines are the best linear fit. (*n* = 332 [SF/Ori], 347 [Dir], and 344 [TF] bins; 11 mice and 67 fields, r: Pearson’s correlation, one-sided test, ∗∗∗∗∗*p* < 10^−10^; ∗∗∗∗*p* < 10^−5^; ∗∗∗*p* < 10^−3^; and ∗*p* < 0.05). Significantly positive r-values indicate that preferences change linearly along the same feature axis as in widefield imaging.

Example fields representing nasal (*field 1*) and near-axial (*field 2*) visual locations had similar numbers of GCaMP8s+ boutons but showed visibly distinct visual feature preferences (Figures 2A and 2B). Boutons viewing nasal locations preferred low spatial frequencies, horizontal orientations, posterior motion, and low temporal frequencies (Figures 2B and 2C, *field 1*). On the other hand, boutons viewing central locations preferred high spatial frequencies, vertical orientations, upward motion, and high temporal frequencies (Figures 2B and 2C, *field 2*). These two example fields suggested that dLGN boutons viewing similar locations represent similar features, whereas those viewing different locations prefer distinct features.

We next quantified similarity in feature preference within the same visual field (*local*) to that across other visual fields (*global*). To do this, we computed the median feature distance between a given bouton and its local and global counterparts (Figure 2D). Subtracting global from local distance (Figure 2E) showed strong negatively skewed distributions for all four feature gradients, indicating that physically proximate boutons prefer similar features as compared to physically distant ones (Figure 2F). This distance-dependent similarity in feature preference continued into individual imaging fields such that individual bouton feature preferences were most like their physically proximate neighbors (Figure S6C); this result is consistent with the idea that nearby boutons likely arise from the same axon.^55^ Taken together, these results indicate that feature preference steadily diverges with inter-bouton separation.

We next asked whether individual boutons gave rise to feature gradients consistent with those obtained with widefield imaging. To this end, we aggregated and spatially binned bouton data across receptive field positions to create a map covering the entire visual field (Figure 2G). These bouton maps resembled those we obtained from widefield imaging: Spatial frequency preference peaked near the optical axis (of the eye) and dropped toward the periphery, orientation preference moved from horizontal to vertical across nasotemporal axes, and temporal frequency preferences grew from ventronasal to dorsotemporal visual locations.

Although 2p feature maps looked more granular and variable than our widefield average maps, gradient analysis across the same characteristic axes defined from widefield imaging showed significant trends for all four visual feature representations (Figures 2H and 2I). Moreover, fields from different mice with approximately similar receptive field positions had feature preferences that matched the comparable position in our feature maps (Figure S7). Also, neuropil signal corrections made no qualitative alteration to the maps and gradients (Figure S8). The presence of similar gradients of feature deviation across individual boutons (2p) and across bouton populations (widefield) indicates that the gradients we observed in the widefield imaging arise primarily from activity of individual boutons.

We also asked whether feature selectivity of dLGN boutons follows the same gradients, even though we did not see such selectivity gradients in our widefield data (Figure S2A). Maps of bouton selectivity binned across visual space also showed small but significant gradients of selectivity for orientation, direction, and temporal frequency along the characteristic gradient axis (Figures S8D and S8E), but no obvious gradient was seen for spatial frequency selectivity. Notably, bouton selectivity for all features was relatively high (all ≥0.5 except DSI >0.2) and within ranges of prior reports^34^^,^^35^^,^^43^^,^^44^^,^^56^ (Figure S8F). Taken together, we conclude that dLGN feature gradients arise from systematic biases in feature preferences of individual dLGN boutons viewing different parts of visual space.

### Functionally and anatomically defined dLGN bouton subsets drive feature organization

Although similar, significant feature gradient trends in our 2p and widefield dataset strongly suggest this organization comes from bouton activity, we could not rule out potential contributions from inherent representational variability^4^^,^^8^^,^^44^ and extrinsic variability such as uneven viral infection. We nevertheless asked if a subset of boutons carried more spatially biased representations. To this end, we sorted boutons according to their functional (responsive vs. selective) or anatomical (layer position) characteristics, re-binned them and recomputed feature gradients.

We started by computing feature maps from either the most responsive boutons for a given stimulus or the most selective ones for a given feature (*see* STAR Methods). Arguments can be made in favor of either bouton population as carrying spatially biased feature representations. For example, more responsive (strongly responding) boutons could better elicit fast and robust postsynaptic responses from cortical neurons. Conversely, more selective (strongly tuned) boutons could better carry biased representations considering efficient coding and decoding. Our analysis points to the first scenario.

**Figure 3:**
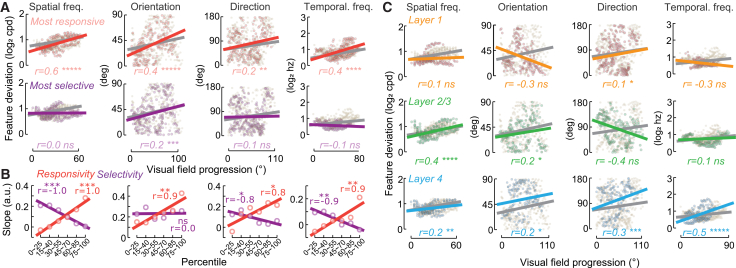
Two-photon imaging reveals functionally and anatomically defined dLGN bouton subsets drive feature gradients (A) Feature deviation versus visual field progression along the previously indicated axes for the top 25% most responsive and selective boutons. Gray dots are bins from all bouton data, taken from Figure 2. Colored dots are bins of selected (most responsive or most selective) boutons. Color and gray lines represent corresponding fits to color or gray points. (B) Slopes (normalized) from the datasets for the indicated quantiles of responsiveness (red) and selectivity (purple). (11 mice, 67 fields, *r* is Pearson’s correlation; ∗∗∗*p* < 10^−3^, ∗∗*p* < 10^−2^, and ∗*p* < 0.05; and ns for not significant, Pearson’s correlation test.). (C) Feature deviation versus visual field progression for boutons in cortical layers 1 (top), 2/3 (middle) and 4 (bottom). Lines are linear fits; *r*, Pearson’s correlation. Significantly positive r-values indicate that preferences change linearly along the feature axis. (11 mice, 48 fields, ∗∗∗∗∗*p* < 10^−10^, ∗∗∗∗*p* < 10^−5^, ∗∗∗*p* < 10^−3^, ∗∗*p* < 10^−2^, and ∗*p* < 0.05; and ns for not significant). Gray dots are bins from all bouton data, taken from Figure 2. Colored dots are bins of selected boutons. Lines represent corresponding fits. XY locations without fields in all 3 layers were excluded.

Gradients for all features were highly significant when computed from the most responsive boutons but were absent when computed from most selective boutons for each feature, except for orientation (Figures 3A and S10). To rule out selection criteria as a driver of our results, we computed gradients across overlapping subsets of bouton responsivity and selectivity for each stimulus. Gradients became increasingly pronounced as we moved across increasingly responsive bouton subsets for each feature (Figure 3B, *red*) but became increasingly weaker when computed from increasingly selective bouton subsets for each feature (Figure 3B, *purple*). These results indicate that dLGN feature gradients are driven by the most responsive (or reliable) dLGN boutons rather than the most feature-selective ones.

Recent work suggests that each cortical layer may be innervated by dLGN neurons carrying a unique mixture of visual information.^43^^,^^57^^,^^58^^,^^59^ In particular, a recent adaptive optics (AO) study showed strong direction- and orientation-tuning of dLGN boutons across cortical layers 1–4.^43^ Thus, we next asked if our feature gradients arise from boutons in a specific cortical layer, or if feature gradients remain the same across the cortical depth. To test this idea, we computed feature gradients from boutons residing in layer 1 (50–100 μm), layer 2/3 (150–250 μm), and layer 4 (350–400 μm; Figures 3C and S11; Figure S9). While gradients in layer 2/3 boutons appeared only for spatial frequency and orientation (Figure 3C, *Layer 2/3*), and gradients appeared only for direction in layer 1 (Figure 3C, *Layer 1*), we saw highly significant gradients for all four features in layer 4 dLGN boutons (Figure 3C, *Layer 4*). While we did not use AO to enhance responses from these deep layer 4 boutons, we note that any blurring due to poor capture of these deep boutons would tend to disrupt the gradients that we observe rather than strengthen them. Moreover, AO enhances neural responses but does not alter bouton population feature tuning.^43^ Taken together, these results indicate that boutons in layer 4 are a major source of dLGN feature gradients.

The strong resemblance of feature gradients in the most responsive boutons and those in layer 4 also suggests why our two-photon maps are more variable than widefield maps (Figure 2H): Every bouton (regardless of responsivity and layer) contributed equally to the feature preference of a given bin in our 2p maps (Figure 2G), distorting their preference. Our widefield maps were likely dominated by boutons providing the strongest signals, as might happen with the densest and most responsive bouton populations in layer 4. Taken together, these results suggest that spatially biased dLGN feature representations are driven by the most responsive boutons and are most pronounced for boutons in layer 4.

### Feature gradients across the retina

Our results so far indicate that dLGN boutons exhibit modest location-dependent biases in their representation of visual features. Where do these biases emerge? We considered three hypotheses: (1) feature biases first arise from the retina and the dLGN simply relays them to cortex; (2) feature biases require V1 feedback to dLGN; and (3) feature organization arises in circuitry local to dLGN.

**Figure 4:**
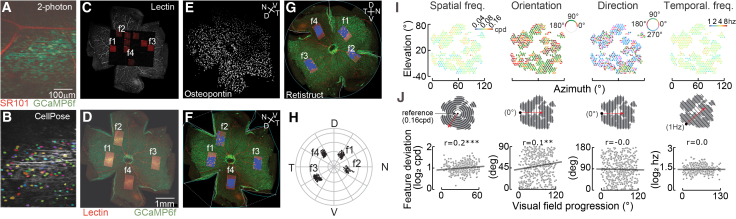
Two-photon imaging of feature preferences in the retina (A) Example two-photon field of GCaMP6f-expressing RGCs counterstained with the blood vessel dye sulforhodamine101 (SR101). Scale bars, 100 μm. (B) Same as A with RGC ROIs. (C) Four two-photon imaged fields (f1–f4) aligned to lectin-stained blood vessels. Additional fields were imaged to capture blood vessel patterns for alignment. (D and E) Two-photon fields mapped onto confocal images of the same retina (D) stained for the dorsotemporal marker, Osteopontin (E). N: nasal; T: temporal; D: dorsal; and T: temporal. Scale bars, 1 mm. (F) Same as D but with ROIs (blue dots) for RGCs. (G) Retistruct transformation of F. (H) RGC ROIs from g in polar coordinates with the optic disc as the origin. (I) Binned RGC preferences in visual field space for spatial frequency, orientation, direction, and temporal frequency from experiments like A–H. (J) Feature deviation versus visual field progression along the indicated axes (insets) for the binned RGC data shown in I. Line is best linear fit; r, Pearson’s correlation. Significantly positive r-values indicate that preferences change linearly along the same feature axis as in widefield imaging (*n* = 367 [SF/Ori], 307 [Dir], and 217 [TF] bins; 5 mice, 50 fields; SF/Ori: 8399 cells, Dir: 6291 cells, TF: 4505 cells, ∗∗∗*p* < 10^−3^ and ∗∗*p* < 10^−2^).

We first tested if dLGN feature organization is inherited from the retina. To do this, we two-photon imaged RGC responses to the same stimuli used for our *in vivo* imaging studies (11,774 identified^60^ RGCs in 9 retinas from 5 mice). Briefly, we explanted retinas from mice in which every RGC expresses GCaMP6f (Vglut2-Cre x Ai95D) and imaged responses from 4 to 9 fields across each retinal surface (Figures 4A–4F). Following imaging, we immunostained retinas with antibodies against osteopontin, which is enriched in the dorsotemporal retina, which let us align retinas along the cardinal anatomical axes of the eye (Figures 4D–4F). Finally, we used standard methods^61^^,^^62^ to map each two-photon imaged field to its immunostained counterpart and transformed these retinal flatmount images back into cups (Figures 4G and 4H).

Next, we spatially binned RGC responses to each visual stimulus as we did for dLGN boutons and mapped each bin to the visual field. Retinal maps of preferred spatial frequency, orientation, direction, and temporal frequency were visibly less organized than those of dLGN boutons (Figure 4I). To quantify this, we measured the feature deviation versus visual field progression along the same axes as we did for dLGN boutons (Figure 4J). We saw a small but significant trend for spatial frequency and orientation, suggesting that dLGN may inherit weak biases for these features from the retina. No significant trends along the characteristic axes were seen for direction and temporal frequency. Overall, these results suggest that most of the dLGN feature gradients arose after retinal output.

### Contribution of cortical feedback to feature maps

**Figure 5:**
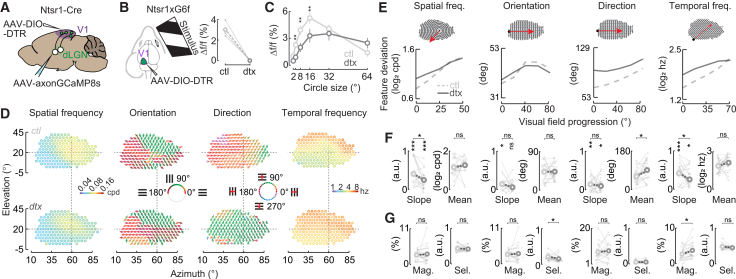
Ablating cortical feedback perturbs dLGN feature maps in widefield imaging (A) Cartoon of an Ntsr1-Cre mouse injected with AAVs carrying axon-GCaMP8s in dLGN and Cre-dependent diphtheria toxin receptor (DTR) in V1. (B) Visual responses of GCaMP6f+ layer 6 corticothalamic (L6) neurons widefield imaged from V1 before and after DTX injection. (C) Widefield axon-GCaMP8s responses to an expanding spot before (ctl) and after DTX treatment (dtx). (∗∗*p* < 0.01, *n* = 18 mice, paired *t* test). (D) Average population bouton feature preferences binned across the visual field before (top) and after DTX (bottom). (E) Feature deviation versus visual field progression along the characteristic axis (inset). Dark gray line (dtx) shows post-ablation average across subjects; light gray, dotted line (ctl), pre-ablation. (F) Slopes (normalized) and mean values of the linear model fits to individual data (dots) in E with the average (open circles). (∗∗∗*p* < 10^−3^, ∗∗*p* < 10^−2^, and ∗*p* < 0.05; and ns not significant, *t* test, *n* = 18). G. Overall response magnitude (Mag) and feature selectivity (Sel) computed over the entire V1 for data in D. Dots, individual data; open circles, condition average. (*n* = 18 mice, ∗*p* < 0.05, paired *t* test).

Cortical feedback sharpens and sculpts dLGN visual responses.^26^^,^^63^ Could these modulatory inputs drive the organization of dLGN feature gradients? To address this, we injected Cre lines (Ntsr1-Cre^64^) granting access to layer 6 corticothalamic cells (L6) with AAVs carrying Cre-dependent diphtheria toxin receptors (DTRs) and labeled dLGN boutons with axon-GCaMP8s (Figure 5A). In this way, dLGN feature maps could be imaged before and after chemogenetic ablation of corticothalamic feedback.

The effects of chemogenetic ablation were confirmed in 3 ways. First, mice expressing both GCaMP6f and DTR showed significant loss of L6 cells 5 days after toxin injection as compared to saline controls (Figures S12A and S12B). Second, we saw complete loss of visual responses in these GCaMP6f+/DTR+ L6 neurons 5 days after DTX injection as compared to controls (Figures 5B and S12C). Third, dLGN bouton population responses to an expanding spot showed significantly altered size tuning after DTX ablation of L6 neurons, consistent with prior reports of feedback modulation of dLGN responses^63^ (Figure 5C).

We next asked how dLGN feature gradients were affected by loss of L6 feedback. Prior to DTX treatment, feature gradients were consistent with our earlier findings (Figure 5D, *top*). Post-ablation maps generally resembled the control data (Figure 5D, *bottom*), but each feature representation showed selective alterations following treatment with DTX (Figures 5D–5G).

While the dLGN spatial frequency gradient remained following DTX treatment, the steepness of this gradient was significantly reduced (Figures 5E and 5F, *Slope*). The overall response magnitude (Figure 5G, *Mag*) and feature selectivity (Figure 5G, *Sel*) remained the same, indicating the gradient reduction was not caused by disrupted or absent responses to this stimulus. dLGN orientation gradients were no longer significant after DTX treatment despite some remaining organization indicated in the average trend (Figures 5E and 5F, *Slope*). Consistent with this, we also saw an overall reduction in orientation selectivity after DTX (Figure 5G). Direction gradients remained significant and did not differ from the control condition (Figures 5E and 5F, *Slope*) but there was a global rotation in the preferred direction relative to their pre-injection counterparts (Figure 5F, *Mean*). There was, however, no associated change in overall response magnitude or selectivity for direction (Figure 5G). Temporal frequency gradients were also retained but were significantly shallower than controls (Figures 5E and 5F, *Slope*). Overall temporal frequency selectivity was not altered, but there was an increase in response magnitude which might be related to temporal precision modulated by cortical feedback^65^ (Figure 5G). None of these changes were observed in saline-injected control mice (Figures S12D–S12F). Taken together, these results suggest that L6 feedback modulates each dLGN feature gradient but does not create them entirely.

## Discussion

We investigated whether mouse dLGN weighs visual features according to location in the visual field. By widefield imaging axon-GCaMP8s+ dLGN boutons across V1, we observed modest location-dependent biases in spatial frequency, orientation, direction, and temporal frequency preference across the visual field (see also Table S1). Two-photon imaging showed that these gradients arose from systematic changes in localized feature preferences of dLGN boutons viewing different visual locations. Gradients were seen in strongly responding boutons rather than strongly selective ones, and those innervating in layer 4 were most like those observed in our widefield data. Retinal two-photon imaging showed weak or absent biases, and loss of corticogeniculate feedback weakened feature gradients but did not abolish them. Taken together, our results demonstrate that dLGN boutons show spatial biases in their visual feature representations, and suggest these biases arise from mechanisms within the dLGN.

### More than just a relay

Growing evidence indicates that dLGN neurons connect to RGC types in specific ways to either enhance or recombine retinal feature signals.^32^^,^^34^^,^^35^^,^^36^^,^^37^^,^^40^^,^^58^^,^^66^ Since dLGN boutons arise from neurons residing in different sectors of dLGN, we reason that our feature gradients reflect systematic changes in the feature preferences of dLGN cells.

Prior work showed diverse feature-selective dLGN neurons^34^^,^^35^^,^^36^^,^^37^^,^^58^^,^^66^ and suggested their preferences arise from synapses formed with specific RGC types.^32^^,^^40^^,^^58^ By inferring patterns of somatic activity from boutons, our results indicate that such wiring specificity can vary depending on where a neuron resides in the dLGN. Importantly, our results predict that neurons residing in the posterior dorsomedial dLGN (nasal visual field) receive stronger input from horizontal orientation-selective RGCs (OS-RGCs)^67^ than those in the anterior ventrolateral region (temporal field), who should connect strongly with vertical OS-RGCs.^28^^,^^67^^,^^68^

RGC innervation biases have been seen with horizontal and vertical OS-RGC projections to the SC whose orientation maps strongly mirror those we see in dLGN.^5^ Given that most RGCs innervating dLGN collateralize to innervate SC^69^ and that mechanisms for retinotopic targeting are shared between both structures,^70^^,^^71^^,^^72^ it is possible that this pattern of OS-RGC axon innervation is also shared. Imaging the OS-RGC boutons across dLGN through cannula-implants^33^ or retrograde tracing from different parts of dLGN into retina followed by type-specific RGC stains could shed more light on this issue. Another possibility is that our gradients reflect differences in the way feature-tuned dLGN neurons innervate V1 (i.e., boutons from horizontally tuned dLGN neurons could be denser in lateral versus medial V1). More work will be needed to investigate these different hypotheses.

### Relationship to visual field specializations in mice and other mammals

Several recent studies have investigated spatially biased feature representation in the early visual pathway. Although many of these reported biases are stronger than our dLGN results, we compare our results to these prior reports later in discussion.

#### Spatial frequency biases

Recent studies indicate that the dorsotemporal and central mouse retina may be specialized for different ethological roles. The dorsotemporal part views the frontal visual field,^73^^,^^74^ receives the least optic flow,^73^ is actively stabilized when mice hunt insects,^73^^,^^75^^,^^76^ and contains RGC types with large receptive field sizes.^20^ On the other hand, the central retina views the lateral visual field,^73^ contains the highest overall RGC density,^77^^,^^78^ and bears a higher density of small receptive field RGC types.^20^^,^^30^^,^^79^^,^^80^ One interpretation is that the dorsotemporal retina is specialized for pursuit of prey whereas central retina is more suited to small or distant objects in the side view.^3^ Such division of labor is seen in retinorecipient SC, where neurons viewing either frontal or lateral space are important for prey pursuit and prey localization respectively.^81^ Our observation that dLGN boutons representing the optical axis prefer high spatial frequencies also fits with this view. It is also similar to primate dLGN, where central representations prefer higher spatial frequencies than peripheral ones,^82^^,^^83^ possibly supporting frontal high-acuity vision in this species. Whether the axial preference for high frequency we saw has a similar behavioral role in mice, possibly for noticing food or prey, is not known.

#### Motion direction biases

Our dLGN representations for motion direction are consistent with prior reports in early visual structures. For example, the tuning of direction selective RGCs align to optic flow patterns over the retinal surface in 4 axes of translation (advance, retreat, rise, fall).^84^ RGCs representing posterior (advance) and upward (fall) were most abundant in these studies and are consistent with other work showing overall bias for posterior versus anterior motion in retinal inputs to dLGN shell,^33^ for horizontal motion in dLGN neurons,^36^^,^^58^ and in cortex.^85^^,^^86^ Collicular representations of the nasal visual field are biased toward anterior motion; whereas its representations more temporal then 30deg away from the midline are biased toward posterior motion^4^ or mostly posterior to reflect the major optic flow by self motion.^8^ Interestingly, RGC boutons in colliculus show posterior motion bias in the nasal visual field and upward bias in the temporal visual field.^5^ A recent study also shows a similar gradient of directional tuning in dLGN neurons that aligns to optic flow dynamics produced by forward motion.^34^^,^^50^ Our dLGN bouton direction maps show similar biases, albeit they are weaker than these prior reports, and were the weakest of the 4 features we examined. One possibility is that our injection strategy poorly labeled neurons in the dLGN shell that receive strong input from retinal direction-selective ganglion cells. Finally, it is important to note that our bouton data from layer 1 could also include boutons originating in the LP, whose neurons are occasionally labeled by axonGCaMP in our approach; it is possible that such contaminating LP boutons diluted the feature gradients we saw in this layer relative to deeper ones.

#### Orientation and temporal frequency biases

Our dLGN orientation feature maps match those observed in the SC^5^^,^^7^ and show horizontal biases at nasal visual locations. They also generally match prior recordings from dLGN neurons,^34^^,^^50^ albeit one of these studies^50^ included frontal visual locations not well sampled by our stimulus monitor. Our bouton biases could arise from a common bias in the way OS-RGCs innervate dLGN and SC, but we also note that both eyes were open during imaging and our screen subtended the binocular zone (*see* STAR Methods). While most dLGN neurons are monocularly driven, it is possible that inputs ipsilateral to our imaging window contribute to this nasal bias, possibly through interneurons which receive more binocular retinal input.^87^ Feature gradients for temporal frequency preference have not been as well investigated as those for orientation and direction.

Finally, we note that while recent imaging studies of V1 neurons show regional variations in population receptive field size,^74^ spatial/temporal frequency preference,^88^ and motion/texture sensitivity,^86^^,^^89^ they do not show the feature gradients we observe in dLGN boutons in V1.

### Cortical feedback enhances dLGN feature maps

V1 can strongly modulate response properties of dLGN neurons.^26^ Studies in several species show various effects including surround modulation^63^^,^^90^^,^^91^ and temporal precision,^65^^,^^92^ alteration in feature preference,^93^^,^^94^^,^^95^ and attentional modulation in the presence of distractors.^96^ Layer 6 neurons exert powerful modulatory control over dLGN through direct projections to this structure and by driving inhibition via dLGN interneurons or via neurons in the thalamic reticular nucleus. Another more speculative possibility is that L6 neurons influence dLGN boutons through translaminar inhibition.^64^ While dLGN boutons do not receive axo-axonic synapses from inhibitory neurons,^26^ there is evidence for GABA-A and GABA-B receptor modulation of thalamic inputs from dLGN and other thalamic nuclei.^97^^,^^98^^,^^99^^,^^100^ Whether such signaling occurs *in vivo* and whether translaminar inhibitory neurons are involved are not known.

While the loss of cortical feedback impacted every dLGN feature map, the effects were specific to each representation. Systematic changes in spatial and temporal frequency preference remained following ablation of layer 6 feedback, but the strength of this gradient was substantially weaker. Azimuthal gradients in orientation preference were lost following ablation and were accompanied by generally weaker orientation selectivity. Gradients for motion direction remained but appeared to rotate overall preference from posterior to vertical motion. Why were these effects feature-specific?

One idea comes from reports in non-human primates showing that corticogeniculate feedback is feature- and stream-specific.^90^^,^^101^ The differing effects we observed in our ablation studies could reflect the loss of a few kinds of L6 feedback cell types. Recent studies suggesting distinct L6 corticothalamic types in mice^102^^,^^103^^,^^104^ strongly support this notion. Such differing L6 types could enable feature-specific enhancements. For example, spatial frequency tuning might be modulated through surround suppression,^63^ while the altered orientation maps and the global rotation effects in motion direction could involve cortical neurons preferring specific features.^93^^,^^94^^,^^95^ Finally, changes in temporal frequency maps can be attributed to corticothalamic control of temporal precision.^65^^,^^92^ Unfortunately, our widefield studies following ablation have insufficient resolution to resolve these bouton-level effects.

Layer-specific two-photon imaging of the same boutons before and after ablation could reveal how feedback affects functionally and anatomically distinct populations of dLGN boutons. However, the small size of boutons makes registration across days challenging. Sparse labeling of dLGN neurons with axon-localized GCaMPs and dLGN-specific Cre driver lines (such as Vipr2-Cre^57^) could offer a way to test this idea in the future.

### Functional specialization and visual ecology

The terrain hypothesis was posited by comparative biologists to explain the visual streak found in the retinae of terrestrial grazing animals whose laterally facing eyes must scan the horizon for potential predators.^105^ This theory posits that an animal’s visual system is adapted to the image statistics of its ecological niche^105^^,^^106^ and offers an evolutionary perspective for visual field specializations in the mouse early visual system.

While most visual features are thought to have an equal likelihood of occurring across a natural scene, some features depart from this kind of invariance.^3^^,^^8^^,^^106^^,^^107^ For example, blue skies fill the upper visual field of many terrestrial vertebrates, whereas their lower field contains earthier tones.^21^ The biases we have shown in our results, along with prior work on visual field specializations, could be viewed as evolutionary adaptations to such visuo-ecological niches. While prior studies speculated that such specializations belong to more ancient parts of the early visual pathway (e.g., retina and SC), our work suggests that specializations are present at the level of dLGN. What purpose could they serve?

One clue could be that dLGN feature maps originate within the most responsive boutons as opposed to the most selective ones. This might reflect the presence of two streams of dLGN input to V1—a strongly driven stream that is biased by visual location, and a more selective stream with weaker spatial bias. The notion of parallel channels has been suggested by recent studies that different dLGN types project to different layers in V1.^43^^,^^57^^,^^58^ Perhaps a “biased” stream enables fast perceptual judgments that capture ethologically salient features along with other subcortical pathways such as SC, whereas the “unbiased” stream is used for versatile scene analysis. Accordingly, it has been proposed that different visual tasks, such as detection versus discrimination, involve extracting visual information from distinct neural populations.^108^^,^^109^^,^^110^ Training mice to detect or discriminate features, such as oriented lines, in different locations across the visual field while providing distractors that either match or mismatch the spatial biases shown by dLGN boutons could shed more light on the behavioral significance of the specializations we have described here.

### Limitations of the study

While our *in vivo* two-photon bouton measurements let us assess aspects of dLGN visual responses without disrupting overlying cortex, this approach has limitations. First, our labeling strategy may have poorly marked direction-selective neurons residing in the shell region of dLGN which could account for the weak direction biases we saw in both widefield and two-photon datasets. Second, the same strategy may have also labeled thalalmic neurons in the LP which could have diluted our feature gradients in superficial cortical layers. Third, while our screen only faced one eye, both eyes were open, which means that neurons in the dLGN on the same side as our imaging window (ipsi) could have contributed to the most nasal parts of our feature gradients. Fourth, tracking the same boutons across days was unfeasible because of their small size, which meant our ablation studies could only be performed at the widefield level. Finally, while these experiments document biases in the dLGN bouton input to V1, future studies will be needed to understand how these signals influence the feature preference of cortical neurons across V1.

## Resource availability

### Lead contact

Further information and requests for resources and reagents should be directed to and will be fulfilled by the Lead Contact, Arjun Krishnaswamy (arjun.krishnaswamy@mcgill.ca).

### Materials availability

This study did not generate new and/or unique materials.

### Data and code availability

• Data to reproduce analysis and main figures have been deposited at https://doi.org/10.5061/dryad.tmpg4f5f9 and are publicly available as of the date of publication. Accession numbers are listed in the key resources table. Accession numbers are listed in the key resources table. Further raw data for this study is available upon reasonable request to the lead contact AK (arjun.krishnaswamy@mcgill.ca).
• Code to analyze data and generate main figures has been deposited at https://github.com/Swamylab/Cha_et_al_Mouse_dLGN_FeatureMaps and is publicly available as of the date of publication. Accession numbers are listed in the key resources table. Further information and requests for resources and reagents should be directed to and will be fulfilled by the lead contact, AK (arjun.krishnaswamy@mcgill.ca).
• Other items: Any additional information required to reanalyze the data reported in this paper is available from the lead contact upon request.

## Acknowledgments

We thank J. Forestell, X. Ma, S. Shariff, J. Lehnert, N. Brake, and F. Jalandoni for helpful comments on the manuscript. This work was supported by grants from the Scottish Rite Charitable Foundation (A.K.), the New Frontiers in Research Fund (A.K. and E.C.), 10.13039/501100000038Natural Sciences and Engineering Research Council of Canada of Canada (A.K. and E.C.), the 10.13039/501100000024Canadian Institutes of Health Research (A.K.). Salary support from the 10.13039/100000879Alfred P. Sloan Foundation, and the Canada Research Chairs Program to A.K.; CONACYT, FRQS, and FRQS VHRN fellowships to A.G.R.O.

## Author contributions

K.C. and A.K., conceived of this study. K.C., A.K., and E.C., aided with instrumentation, analysis, experiments, writing, and making figures. A.G.R.O., performed retinal experiments. R.S., provided experimental advice and comments on the manuscript.

## Declaration of interests

We, the authors and our immediate family members, have no financial interests to declare. We, the authors and our immediate family members, have no positions to declare and are not members of the journal’s advisory board. We, the authors and our immediate family members, have no related patent applications or registrations to declare.

## STAR★Methods

### Key resources table

**Table:** 

REAGENT or RESOURCE	SOURCE	IDENTIFIER
**Antibodies**		

chicken anti-GFP (1:1000)	Abcam	RRID:AB_300798
goat anti-osteopontin (1:1000)	R&D Systems	RRID:AB_2194992

**Bacterial and virus strains**		

AAV2/9-hSyn-axon-jGCaMP8s-P2A-mRuby3	Neurophotonics	SKU: 1879-aav9
AAV2/retro-CAG-flex-DTR	Neurophotonics	SKU: 1482-aavretro

**Chemicals, peptides, and recombinant proteins**		

Alexa Fluor 488	Cedarlane	RRID:AB_2340375
Cy3	MilliporeSigma	RRID:AB_92570
Isolectin	Fisher Scientific	RRID:SCR_014365
Sulforhodamine 101 (SR101)	MilliporeSigma	Cat# S7635
PBS 10x	MilliporeSigma	Cat# 11666789001
Triton X-100	MilliporeSigma	Cat# X100
Ames solution	MilliporeSigma	Cat# A1420
Donkey serum	MilliporeSigma	Cat# S30-M
Diphtheria toxin	MilliporeSigma	Cat# D0564

**Deposited data**		

Raw data to reproduce analysis and main figures	Dryad	https://doi.org/10.5061/dryad.tmpg4f5f9
Code to reproduce analysis and main figures	Github	https://github.com/Swamylab/Cha_et_al_Mouse_dLGN_FeatureMaps

**Experimental models: Organisms/strains**		

Mouse: Ntsr1-Cre	The Jackson Laboratory	RRID:IMSR_JAX:03026
Mouse: VGluT2-Cre	The Jackson Laboratory	RRID:IMSR_JAX: 016963
Mouse: Rosa26-CAG-GCaMP6f	The Jackson Laboratory	RRID: IMSR_JAX:028865

**Software and algorithms**		

Suite2p	Pachitariu et al.^54^	https://github.com/MouseLand/suite2p
CellPose	Stringer et al.^60^	https://www.cellpose.org/
Retistruct	Sterratt et al.^61^	https://github.com/davidcsterratt/retistruct
ImageJ	Fiji	RRID:SCR_002285
R	r-project	RRID:SCR_001905
Python	Python^TM^	RRID:SCR_008394
ScanImage	Vidrio Technologies	RRID:SCR_014307
MATLAB	Mathworks	RRID: SCR_001622
Psychtoolbox	https://www.psychtoolbox.net/	RRID: SCR_002881
Code to analyze data and figures	https://github.com/Swamylab/Cha_et_al_Mouse_dLGN_FeatureMaps	N/A

**Other**		

Neuros syringe	Hamilton	Cat# 65460–03
Microsyringe pump	World Precision Instrument	Cat# UMP3-4
Filter paper	MilliporeSigma	Cat# HABG01300
DLP light crafter	Texas Instruments	Cat# 595-DLPLCR4500EVM
Mounted LED (470 nm)	Thorlabs	Cat# M470L5
GFP emission filter	Thorlabs	Cat# MDF-GFP
Mai Tai HP Ti:Saphire Ultrafast laser	Spectra-Physics, CA, USA	S/*N* 20405
Femto fixed 920 nm laser	MPB communications	N/A

### Experimental model and study participant details

#### Animals

Adult (2-4 month) Male and female wildtype C57BL/6, Ntsr1-Cre (RRID:IMSR_JAX:030264) and VGluT2-Cre mice (RRID:IMSR_JAX: 016963) were used in this study. For widefield experiments, we used adult (2-4month) 18 wildtype animals (14 males and 4 females) and 18 Ntsr1-Cre animals (6 males and 12 females); effects of sex on our results were not analyzed. Eleven mice were used in two-photon imaging. Ten of them were included in the 36 mice used for widefield imaging. Rosa26-CAG-GCaMP6f (RRID: IMSR_JAX:028865) mice crossed with VGluT2-Cre mice for experiments with RGCs and Ntsr1-Cre mice for testing the functional efficacy of chemogenetic ablation. All surgical and experimental procedures were in accordance with the rules and regulations established by the Canadian Council on Animal Care, and protocols were approved by the Animal Care Committee at McGill University (Animal Use Protocol # MCGL-7889).

### Method details

#### Virus and diphtheria toxin injections

Mice were anesthetized during virus injections at 2% isoflurane (4% for induction). A Neuros syringe (Hamilton) was used to deliver AAV-hSyn-axon-jGCaMP8s-P2A-mRuby3 (Neurophotonics, Laval) to dLGN. To prevent spill-over into the visual cortex, we approached dLGN from the top of somatosensory area (-2.1mm lateral and 0.8mm posterior to the bregma) at a 30-degree angle, targeting 2.1mm lateral and 2.6mm posterior to the bregma and 3.1 mm below the skull. Injection was performed slowly (200-250nl for 3-4 minutes) using a microinjector (World Precision Instruments) and the needle was kept in position for >5 minutes before it was retracted. In case of LP injection for Figure S1, the same angled approach was applied but 0.5 or 1 mm medially to the dLGN-targeted coordinate (i.e., 1.6mm or 1.1mm lateral to the bregma).

Using the same setup, AAV2/retro-CAG-flex-DTR (Neurophotonics) was delivered to the left V1 in Ntsr1-Cre mice. For wide expression over the V1, injection was performed at 3 sites which are 0.5∼1mm from the center of the V1 (2.5mm lateral and 0.1 anterior to the lambda) 600μm below the dura. A volume of 150nl was delivered to each site. Usually, these injections were done at cranial window implantation. Diphtheria toxin (Sigma D0564) was administered intraperitoneally (100 ng/g body weight) to mice expressing DTR after their control imaging session.

#### Headplate and cranial window implantations

Mice were induced with 4% isoflurane and analgesics (carprofen at 5cc/g and topical lidocaine) given before surgery. Anesthesia was maintained using 2% isoflurane. After surgery, the animal was placed back in its home cage and provided with carprofen for the following 2 days.

Headplates were implanted after dLGN injections over the exposed skull around the left V1 using dental cement. For this, a circle was marked for the placement of cranial window and dental cement was applied to the surrounding area. A headplate with a circular hole was placed on top of the cement and then additional dental cement was applied. After recovery, the animal underwent cranial window surgery. A 4 mm-diameter craniotomy was made over the left V1 using a dental drill. After removing the skull flap, a round cover glass was gently placed in the craniotomy and sealed with cyanoacrylate glue while kept in place with a small rod attached to a micromanipulator.

#### Histology

Euthanasia of mice by isoflurane overdose and perfusion with phosphate buffered saline (PBS) was followed by brain extraction. The brains were postfixed in 4% (w/v) paraformaldehyde (PFA) overnight. A tissue slice (Compresstome) was used to collect 150- or 200-um thick coronal sections of the brain. For immunostaining, the brain sections were incubated in blocking buffer (10% normal donkey serum, 0.4% Triton X-100 in PBS) for 1-2 hours, followed by incubation in primary antibodies at 4°C for 7 days, and secondary antibodies at 4°C overnight. Sections were imaged under a Zeiss confocal microscope where 405nm wavelength was used for excitation.

Two-photon imaged retinas were dissected and fixed for 45 min in chilled 4% (w/v) PFA, 20 min in methanol and 20 min in acetone. For immunostaining, whole-mounts were incubated with blocking solution (4% normal donkey serum, 0.4% Triton-X-100 in PBS) for 2 hours, followed by incubation with primary antibodies overnight at 4°C. Tissue was then washed with PBS and incubated in secondary antibodies for 2 hours at room temperature. Images of stained tissue were acquired on a Zeiss LSM-710 inverted confocal microscope at a resolution of 512 × 512 pixels with a 0.3 μm step size.

Antibodies used were as follows: chicken anti-GFP (1:1000, Abcam, Cambridge, UK; RRID:AB_300798) and goat anti-osteopontin (1:1000, R&D Systems, Minneapolis, MN; RRID:AB_2194992) and rabbit anti-DsRed (1:1000, Clontech Laboratories; RRID:AB_10013483). Secondary antibodies were conjugated to Alexa Fluor 488 (Cedarlane, Ontario, CA; RRID:AB_2340375) or Cy3 (MilliporeSigma; RRID:AB_92570; RRID:AB_92588). Isolectin (Fisher Scientific, Waltham, MA; RRID:SCR_014365) was incubated along with the secondary antibodies.

#### Visual stimulation

Visual stimuli were presented on an LED screen (INNOCN, 34.4 cm × 19.4 cm) placed perpendicularly to the optical axis of the right eye of the mouse. The center of the screen was located 10 cm away from the optical axis and the left end of the screen was aligned to the midline of the subject. This resulted in a coverage of 120 degrees in azimuth and 80 degrees in elevation. These screens were selected to minimize viewing angle effects on luminance because their large viewing angle (170 degrees) is more than double the angle between the mouse’s optical axis and the screen edge (60 degrees). The red color channel of the monitor was disabled, and all stimuli were presented on a linearized monochromic scale out of a uniform background at the midpoint luminance (grey). In the experiments for Figure S4, spherical correction was applied as previously reported^111^ using Psychtoolbox (Psychtoolbox-3 - DisplayUndistortionLabRiggerMouseStim).

Visual stimuli were implemented with Psychtoolbox and controlled by in-house software run on MATLAB. For retinotopic mapping, a white or checkerboard bar swept across the screen either horizontally or vertically at the speed of 10 degrees/second. The thickness of the bar was 10 degrees. Each direction was repeated 40 times for widefield imaging and 10 times for 2-photon imaging. For size tuning, a white or black disc was presented at the center of the screen with the diameter randomly chosen each trial from a set of 2, 4, 8, 16, 32 and 64 degrees. Each of 120 presentations was 2-second long and flanked by grey background screen (1 second before and 2 seconds after presentation).

For feature tuning, static gratings, drifting gratings and luminance-oscillations were presented for 2 seconds each trial subtending the full screen (Figure S1E). In static grating experiments, spatial frequency was randomly chosen out of 0.04, 0.08 and 0.16 cycles/degree, and orientation was drawn from 0, 45, 90 and 135 degrees. Spatial phase was also randomly chosen from 0, 90, 180 and 270 degrees. Typically, 240 trials were given for widefield imaging and 120 for 2-photon imaging. In drifting grating experiments, one of 8 directions (0, 45, 90, 135, 180, 225, 270, and 315 degrees) was drawn each trial. The spatial frequency was set at 0.05 cycles/degree and the grating drifted at the speed of 60 degrees/second (3 Hz). Typically, 160 trials were given in widefield imaging and 80 for 2-photon imaging. In luminance-oscillation experiments, spatially uniform luminance changed sinusoidally in time for 2 seconds each trial at one of 4 frequencies (1, 2, 4, and 8 hertz). The temporal phase was either 0 or 180 degrees, randomly drawn each trial. Typically, 160 trials were delivered in widefield imaging and 80 for 2-photon imaging.

We performed retinal stimulation as described previously.^62^^,^^112^^,^^113^^,^^114^ Briefly, a DLP light crafter (Texas Instruments, Dallas, TX) was used to project dichromatic (405nm, 520 nm) visual stimuli through a custom lens assembly that steered stimulus patterns into the back of a 16× objective. All visual stimuli were displayed with a background intensity set to 1 × 104 R∗/rod/s. The projector LED and the scan retrace of the two-photon microscope was synchronized using custom electronics.^115^ The 3 feature stimulus sets (static gratings, drifting gratings and full-field luminance oscillations) were presented with a spatial conversion at 30um per degree. Each stimulus was presented in a fixed order for 1 second twice. A 1-second-long gray background period was given between presentations.

#### *In vivo* widefield and two-photon imaging

For widefield imaging, we used a custom-built epi-fluorescent microscope to capture the visual cortex (V1) through a cranial window at a 10 Hz sampling rate using custom code written in LabVIEW (National Instruments). Mice were lightly sedated (0.5-1% isoflurane) on a semi-cylindrical platform with its headplate affixed to a metal holder. Acquired images covered a 4 mm×4 mm field of view at a 180×180 resolution. Camera acquisition parameters and LED illumination power for both autofluorescent control and axonGCaMP8s mice were identical.

For two-photon imaging, cranial-window implanted mice were placed under a custom-built galvo-galvo-resonant two-photon microscope and lightly sedated. A 16x NA .8 water immersion objective (Olympus) was lowered atop the cranial window to focus into V1. The fluorophores were excited at 920 nm using a Mai-Tai (Newport) equipped with dispersion compensation and laser power at the sample plane was typically 5-15 mW. A 280×280 um^2^ field of view was scanned at 45Hz as a series of 512×512 images. Imaging depth was measured from the pia which was identified by illuminating the field at 1024 nm. Imaging field locations were determined by real-time observing active boutons responding to a bar sweeping across the visual field. Once an imaging field location is decided, it was attempted to image three depths of layer 1 (50-100 μm), layer 2/3 (150-250 μm), and layer 4 (300-350 μm) along the same column. Collecting data from all 3 layers was possible in sixteen field locations (48 fields), which were included in the layer-specific feature analysis. An additional 19 fields were included in all the other analyses.

#### Two-photon imaging of retinal ganglion cells

Two-photon imaging was performed as previously described.^62^^,^^112^^,^^113^^,^^114^^,^^116^^,^^117^^,^^118^ Briefly, mice were dark adapted for at least 2 hours, euthanized, and then retinas rapidly dissected under infrared illumination into oxygenated Ames solution (95% O2, 5% CO2; MilliporeSigma, A1420). Next, retinas were mounted onto a filter paper (MilliporeSigma, HABG01300) with the RGC layer facing up, placed in a recording chamber, mounted on the stage of a custom-built two-photon microscope, and perfused with oxygenated Ames solution warmed to 32–34°C. Responses of GCaMP6f+ RGCs to visual stimuli delivered through the objective were imaged at 920 nm (MPB Femto fixed 920nm laser) and collected at an imaging rate of 45 Hz. Each image plane (483.5×483.5 μm) of the movie contained GCaMP fluorescence, SR101 fluorescence, stage coordinates, and visual stimulus synchronization pulses to permit offline analysis. A few microliters of sulphorhodamine 101 (SR101, 2 mg/mL, MilliporeSigma, S7635) was added to the recording chamber to label blood vessels and a map of the main blood vessels emanating from the optic disk acquired for post hoc image registration. Data acquisition was performed with ScanImage synced to the visual stimulus using a photodiode sync pulse and handshake pulse using custom made code in MATLAB. Following recording, retinas were fixed, immunostained and imaged as described above.

#### Time-series data processing for *in vivo* dLGN bouton imaging data

The following time-series processing was applied to each pixel of widefield imaging data and each ROI of cortical two-photon imaging data. Suite2p software^54^ was used to define ROIs (519,603 in total) and extract their time-series data from 2-photon images. Time-lapsed image intensity values of each trial were first resampled at 5 Hz and were converted to proportion of change from a baseline, ΔF/F = (F - F_0_)/F_0_. The baseline, F_0,_ was computed from the average values of a 1-second-long grey background period that preceded stimulus onset each trial. These values underwent a Gaussian smoothing to increase reliability of the baseline estimation and alleviate the effect of transient changes. Specifically, a Gaussian kernel with sigma = ∼1 trial was applied to the series of baseline values from each trial. In the case of bar stimuli for retinotopy, the time series data were concatenated across trials for Fourier analysis. For the other stimuli, the response for each trial was obtained by averaging ΔF/F over the period of stimulus presentation (for 2 seconds after stimulus onset). Most of the data presented in this study was not Neuropil corrected^54^; parallel analysis with correction shows little qualitative difference from our main data (Figure S8).

#### Data processing of retinal imaging data

ROIs were defined using CellPose,^60^ and MATLAB was used to extract time series data for each ROI. The signals were smoothed to remove noise with moving mean of 10 frames and expressed as ΔF/F, where the mean of the peristimulus baseline was used to calculate the difference. The response for each stimulus presentation was obtained by taking the maximum value of the calcium signal during the one second of stimulus presentation. Responsiveness of ROIs were evaluated for each of the three stimulus sets as in cortical image data analysis (See above) except the response reliability was computed as the maximum response divided by the standard deviation of baseline activity. Only ROIs whose response is above 3 standard deviations were included in the feature analysis.

Retinas were immunostained after two-photon calcium imaging and image registration was performed as previously described.^62^ Briefly, the blood vessel image of each recorded field was stitched into a single image using custom MATLAB scripts, while a confocal image was acquired for the same field after staining. Following scaling, rotation, and translation the two images were overlayed to match sulforhodamine101 and lectin blood vessel patterns. Next, fine blood vessel morphology was used as inputs for landmark correspondence in Fiji to register each two-photon field into a whole-mount retina confocal image. The whole-mounted retina was orientated in the D-V-T-N axis using the density of alpha RGCs as a reference (higher density in the D-T region) which were labelled with an osteopontin staining. The orientation of the stimuli presented during two-photon imaging was also orientated using alpha RGC density as reference.

Following registration, each ROI obtained was mapped into the whole-mount retina space using the linear transformation matrix obtained from Fiji’s landmark correspondence. These ROIs were projected from a flattened retina into a reconstructed standard spherical retina space using the Retistruct package for azimuthal equilateral projections. All ROIs from 9 retinas were merged in a single projection, where left retinas were mirrored into the right retina space. Polar coordinates given by Retistruct were then transformed to cartesian coordinates of visual field positions centered at the optical axis to match the coordinate system of the *in vivo* data.

#### Widefield retinotopy mapping and image alignment

Each pixel of widefield image data from retinotopy experiments underwent fast Fourier transform to estimate the phase of the periodic responses to bar positions sweeping across the screen horizontally or vertically (Figure S1F). The azimuth and elevation phase representations in the image space were used to compute vector field gradients to find the boundary of V1 in each mouse.

A cortical region that is responsive to the center of the screen (the optical axis of the eye) was defined for alignment of image data across individual mice in the following way. Single-trial responses to a small circular stimulus (4 or 8 degrees in diameter) presented at the center of the screen were converted to t-values and the t-map was smoothed (Gaussian filter with a sigma value of 4 pixels). A blob of spatially clustered pixels with the highest 0.2 % t-values (∼65 pixels) was defined as the cortical representation of the optical axis, whose center was used as a reference point to be aligned to across mice. Image translation was performed to align the images at the reference point. We refer to the aligned image space as standard image space in which all individual mouse datasets reside as a result of the alignment.

After the alignment, the primary visual cortex was demarcated by selecting the pixels that were indicated as the primary visual cortex in at least 18 of the 36 mice (P(V1) ≥0.5). This region we refer to as standard V1 is used for all 36 individual datasets. Further data analysis was done only in the pixels of this region.

In the cortical ablation experiments, within-subject image registration was performed using MATLAB image processing toolbox to align datasets acquired before and after ablation. Mean images from the first run of the retinotopy imaging were used as a reference image.

### Quantification and statistical analysis

#### Confocal image analysis

Linescans and cell counts were quantified using custom ImageJ and MATLAB scripts as previously.^62^^,^^114^ Briefly, linescans of pixel intensity were normalized to the maximum signal intensity. These normalized traces from multiple brain sections were aligned and averaged to obtain the mean intensity ± SEM across conditions.

#### Bouton ROI selection

After Suite2p selected tentative bouton ROIs, responsiveness of the Suite2p ROIs was evaluated for each of the three stimulus sets (static gratings, drifting gratings, and full-field luminance oscillations). Single-trial responses of the preferred stimulus condition, which was determined by the largest average response of all conditions, were t-tested and ROIs that passed a criterion of *p* < 0.01 were selected for further analysis. The selection was performed for each stimulus set so that different sets of ROIs were chosen for analysis (Figure S6A). The t-scores of the preferred response of these ROIs were also used in the analysis for responsive quantiles.

The second criterion was the deviation of receptive field position from the median receptive field position of the boutons in the same imaging field. We examined the overall distribution of the deviation and set the cutoff value at 95% which corresponded to 30.1 degrees (Figure S6B). Therefore, ROIs whose receptive field position was away from the population median of the field farther than 30.1 degrees were discarded. Cortical dendrites and axons were not labelled with our angled injection approach so no neuropil correction was performed.

#### Estimation of receptive field position

Receptive field position was estimated in the same manner for each pixel of widefield data and ROIs of *in vivo* two-photon data. The position of the bars sweeping across the screen either horizontally or vertically was resampled at the same rate as the time series (5 Hz) and the average across trials was approximated on a grid of every 2.5 degrees. The fluorescence time series data were also averaged across trials over one condition (e.g., horizontal sweeps), which yielded a ‘position tuning’ function. The position tuning function was interpolated at every 0.01 degrees and the peak position was assigned as receptive field position. Azimuth and elevation were estimated separately.

After estimation of pixelwise (i.e., population) receptive field position in widefield data of each mouse, the median across the individuals were computed in the standard image space. There was a systematic shift in these estimates indicated by the receptive field position of the screen center region in widefield data, which was (6.3, 6.5). This shift, presumably caused by the delay in fluorescence responses, was corrected by subtracting the offset values. In the case of widefield data, the resultant azimuth and elevation values, we refer to as standard azimuth and elevation, were assigned back to individual data.

Drawing the boundary of the binocular zone was hard because the estimated receptive field positions are limited by the screen edges and averaging those estimates near the edges shifts the average towards the center of the screen. It is also possible that cortical magnification distorts the nasal versus temporal field. However, as the nasal screen edge (0 deg) was aligned to the midline of the animal and the screen center to the mouse optical axis (60 deg), we estimate the binocular zone subtends 0-20deg.

#### Global vs. local feature distance analysis for individual bouton ROI

To evaluate local homogeneity of feature preference, we computed local and global feature distances for each bouton ROI. Local feature distance was defined as preferred feature difference between a given bouton ROI and the other boutons within the imaging field. Global feature distance was defined as preferred feature difference between a given bouton ROI and bouton ROIs in the other fields. Computing the median values of these two sets of distance values yielded a paired sample to measure local (in)homogeneity. The paired sample underwent a sided sign test against a null hypothesis that boutons have the equal or larger median feature distances from their within-field members than from those outside the field.

#### Computation of preferred feature and feature selectivity

The following analysis was applied to each pixel of widefield data and each bouton ROI in 2-photon data, similarly to a previous study.^57^ Preferred features were estimated using a vector sum formula, $\hat{\theta }=∠\sum r_{k}{\mathrm{e}}^{i\theta_{k}}$, where r_k_ and θ_k_ are the average response and the feature value for stimulus condition k. For orientation, θ_k_ was 2 times of the stimulus orientation angle and $\hat{\theta }$ was multiplied by 0.5 as preferred orientation. In the case of spatial frequency and orientation, since the 2 feature domains were combined in a static grating stimulus, the best stimulus parameter of the other domain was determined first. For example, in estimation of preferred orientation, the best spatial frequency for a pixel or an ROI was first found and the responses to orientations were chosen among the responses to that spatial frequency. The same principle was applied to estimating preferred spatial frequency.

For spatial frequency and temporal frequency, the original feature values were converted to angular values on a semi-circle. In other words, spatial frequencies 0.04, 0.08 and 0.16 cpd were log-scaled and evenly placed in the semi-circular space, resulting in 0, π/2 and π. In the same way, temporal frequencies 1, 2, 4, and 8 Hz resulted in 0, π/3, 2π/3 and π, respectively. Vector averaging on a semi-circle leads to compression of the resulting values towards the centroid. We overcame this by adjusting r_i_ so that the minimum is set to 0, which allowed the preferred feature value to be distributed across the full range of the feature space. After the vector averaging, $\hat{\theta }$ on the semi-circle was converted to the native frequency values by inverse mapping. Feature selectivity was computed by utilizing the circular operation as well: $\hat{\rho }=\left|\sum r_{k}{\mathrm{e}}^{i\theta_{k}}\right|/\sum \left|r_{k}\right|$.

In averaging preferred feature values either across animals or within a spatial bin, spatial frequency and temporal frequency were log-transformed, averaged and transformed back to the native frequency scale; and orientation and direction were averaged using the vector sum method described above.

#### Spatial binning

Spatial binning was applied to create feature maps in the visual field space and feature gradient analysis. Pixelwise data from widefield imaging were spatially binned based on receptive field positions into a hexagonal grid. The radius of the bin was set as 2.5 degrees. The same grid was applied to *in vivo* 2-photon ROIs. In the case of dLGN bouton ROIs, only the bins with at least 30 ROIs were used for further analysis. In the case of two-photon data from the retina, only the bins with at least 10 ROIs were included.

#### Feature gradient models

Systematic feature preference changes were captured as gradients of the changes along characteristic axes. Of note, this approach is analogous to previous studies that used consistent positive changes across positional segments to quantify similar phenomena; but quantifying them as a single slope measure is a more stringent and succinct method.

Feature gradient axes were determined by observation of the visual field maps of preferred features and did not involve numerical optimization. The gradient axis is expressed as follows: (1) for spatial frequency, $\chi =\sqrt{x^{2}+y^{2}}$; (2) for orientation, *χ*=*x*, (3) for direction: *χ*=*x*, and (4) for temporal frequency, $\chi =\left(x+y\right)/\sqrt{2}$, where x is azimuth and y is elevation. The origin of the x-y coordinate system corresponded to the optical axis of the eye.

The gradient model is generally described as *g*(*χ*)=*β*_0_+*χβ*_1_+*ε*, where *g*(*χ*)is feature deviation from a reference value at *χ*, *β*_0_ and *β*_1_ are the intercept and the slope of a linear fit, and *ε* is an error term. The feature deviation was defined for each domain as follows (Figure S2B): (1) for spatial frequency, *g*(*χ*)=|*log*_2_θ(*χ*)-*log*_2_0.16| where θ(*χ*) is spatial frequency at *χ*; (2) for orientation, *g*(*χ*)=|angdiff(θ(*χ*), 0)| where angdiff is angular difference; (3) for direction, *g*(*χ*)=|angdiff(θ(*χ*), 0)|; and (4) for temporal frequency, *g*(*χ*)=|*log*_2_θ(*χ*)-*log*_2_1|. The receptive field positions of widefield data were transformed to *χ* and the feature values were transformed to *g*, so a linear function could be fit to estimate *β*s for each mouse, using the least-squares method (MATLAB polyfit function). Slope estimates *β*_1_ were obtained in each mouse (Figures S2C–S2E) and underwent a t-test. They were normalized for visualization in Figures 1H and 5F so that the slope value would be ranged in [-1, 1]. For 2-photon imaging data, Pearson’s correlation was computed to test the linear relationship between *χ* and *g*.

For widefield data, statistical inferences were made, by t-tests, for the mouse population as we tested the slope values of a sample of individual mice against the null hypothesis that the mouse population does not have a positive slope, or gradient, along the characteristic gradient axis. For 2p data, the inferences were made for the bouton aggregates for the given mouse group against the null hypothesis that aggregated bouton feature preferences do not comply with a linear relationship between *χ* and *g*, i.e., the gradient model.

#### Summary statistics

Table S1 provides statistical significance and effect size for all figures. All statistical tests are reported in the figure legends and *p*-values indicated according to asterisks, where generally: ∗ *p* <0.05, ∗∗ *p* <0.01, ∗∗∗ *p* <0.001, ∗∗∗∗ *p* <0.0001. Some very small *p*-values are reported in the figure and associated legend without asterisks.
