# Supplementary material for: Spatial biases in visual feature representation of mouse dorsal lateral geniculate nucleus boutons
Source: iScience. 2026 Aug 8;29(8):117088. doi: 10.1016/j.isci.2026.117088 (PMC13476559; doi:10.1016/j.isci.2026.117088)
Supplement: Supplementary file 1 [file mmc1.pdf]

iScience, Volume *29*

Supplemental information

Spatial biases in visual feature representation

# of mouse dorsal lateral geniculate nucleus boutons

Kuwook Cha, Aline Giselle Rangel Olguin, Reza Sharif-Naeini, Erik P. Cook, and Arjun Krishnaswamy

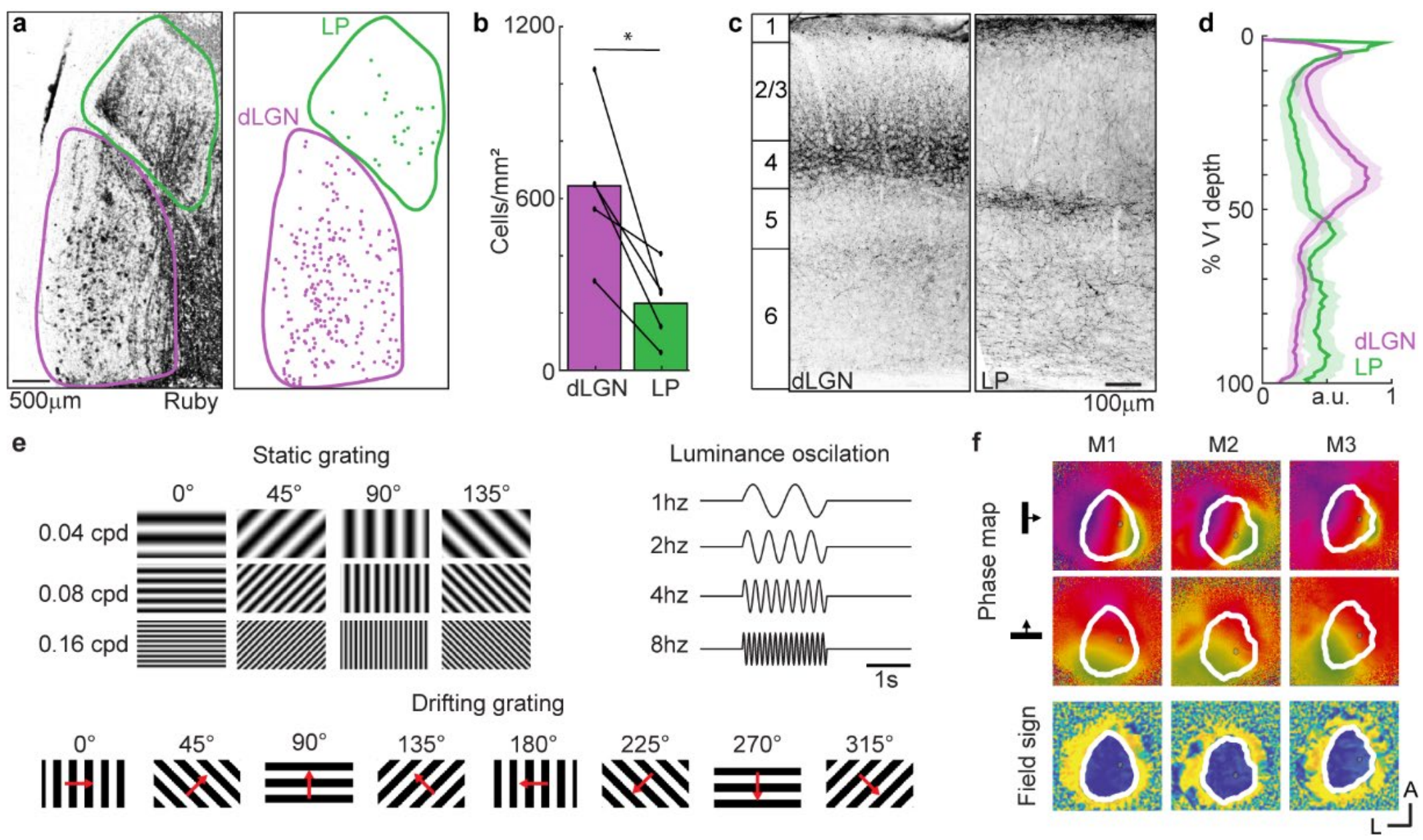

Figure S1. Injection strategy validation, feature stimulus sets and retinotopic mapping. a. Example cross section taken from a mouse injected with axon-localized GCaMP8s in dLGN stained for the co-expressed Ruby reporter protein (left) and a diagram of the same cross section with positions of dLGN and LP somata through the stack indicated. **b.** Quantification of dLGN and LP cell density in experiments like those shown in c (n= 5 animals). **c.** Example cross sections through the visual cortex (V1) of mice injected with axonGCaMP8s in dLGN or in LP. These two thalamic nuclei exhibit very different projection patterns in V1. **d.** Average line-scans of fluorescence taken across cortical depth from experiments like those shown in c. **e.** The static grating set (top right) included 12 conditions of 3 spatial frequencies (0.04, 0.08 and 0.16 cpd) x 4 orientations (0, 45, 90 and 135°) conditions. Spatial phase was randomly drawn out of 4 (0, 90, 180, and 270°) each presentation. The drifting grating set (bottom) included conditions of 8 directions (0, 45, 90, 135, 180, 225, 270, and 315°). The grating drifted at 60°/s and the initial phase was randomly drawn from 0, 90, 180, and 270°. The luminance oscillation set included 4 conditions of temporal frequencies (1, 2, 4 and 8 Hz) and the initial phase was randomly drawn from 0 and 180°. **f.** Retinotopic phase maps and field sign maps of 3 example mice. White boundary represent V1 defined by field signs, and small gray blobs represent regions responsive to the center of the screen. All image data across subjects were aligned at the optical axis (small gray blob).

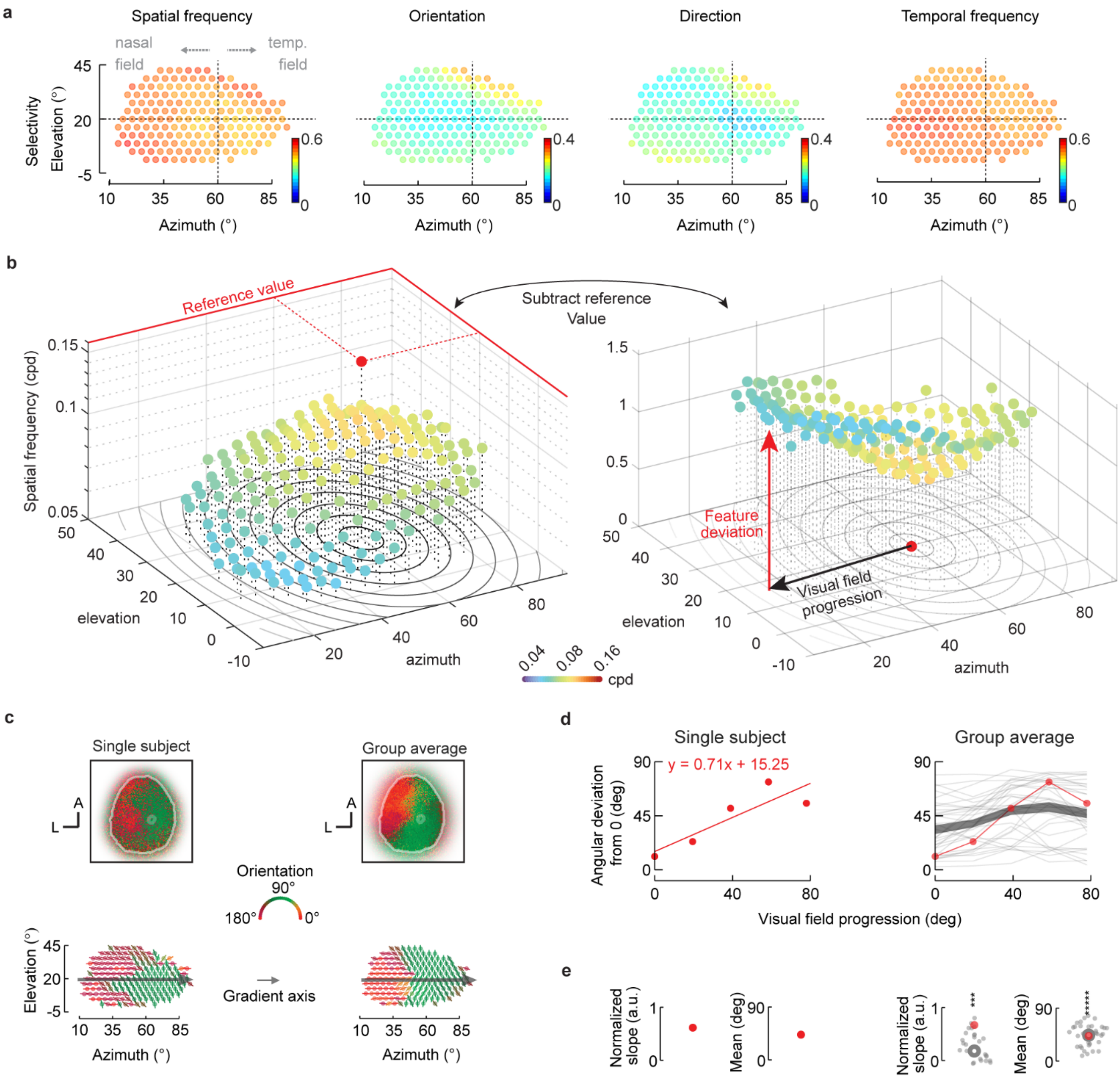

Figure S2. Selectivity maps and gradient analysis. a. Maps of selectivity computed from widefield imaging and transformed into 2.5^{0} hexagonal bins across visual space for the indicated visual feature (n = 36 mice). **b.** Example of how feature gradients are computed. 3D scatter plot of spatial frequency preference binned across the visual field. A reference value is deducted from each bin to transform the map into a gradient plot allowing measure of visual field progression (black line) and feature deviation (red line). **c.** Example (left) and average (right) orientation maps on visual cortex (top) and transformed into the visual field (bottom). Arrow indicates the nasotemporal gradient axis for orientation. Anterior (A) and lateral (L) indicated. **d.** Left: single subject data binned across the gradient axis (red dots) and fit with a line. Right: the same binned data also shown with other subjects (red and gray thin lines) and group average (thick line mean ± SEM). **e.** The slope and the mean estimates of the linear model in a single subject (left) and plotted alongside all animals (right). Average of slope and mean estimates indicated by large gray ring.

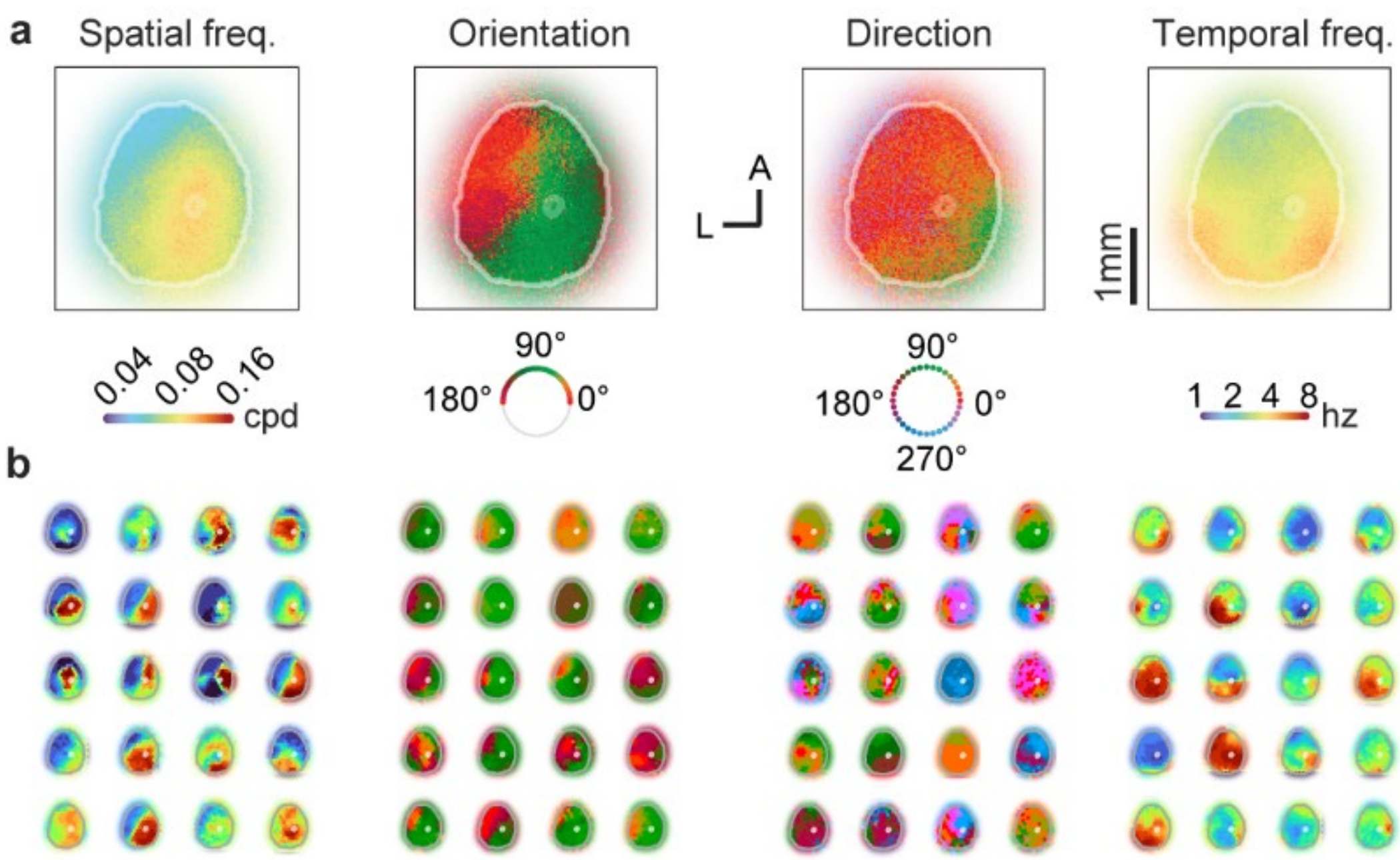

Figure S3. Variability in individual mouse widefield maps. a. top: Average preference of dLGN population boutons across V1 spatial frequency, orientation, direction, and temporal frequency. b. Example maps of bouton preference for spatial frequency, orientation, direction and temporal frequency taken from 20 individual mice. Maps in Panel a were reproduced from Manuscript Figure 1e. Anterior (A) and lateral (L) indicated.

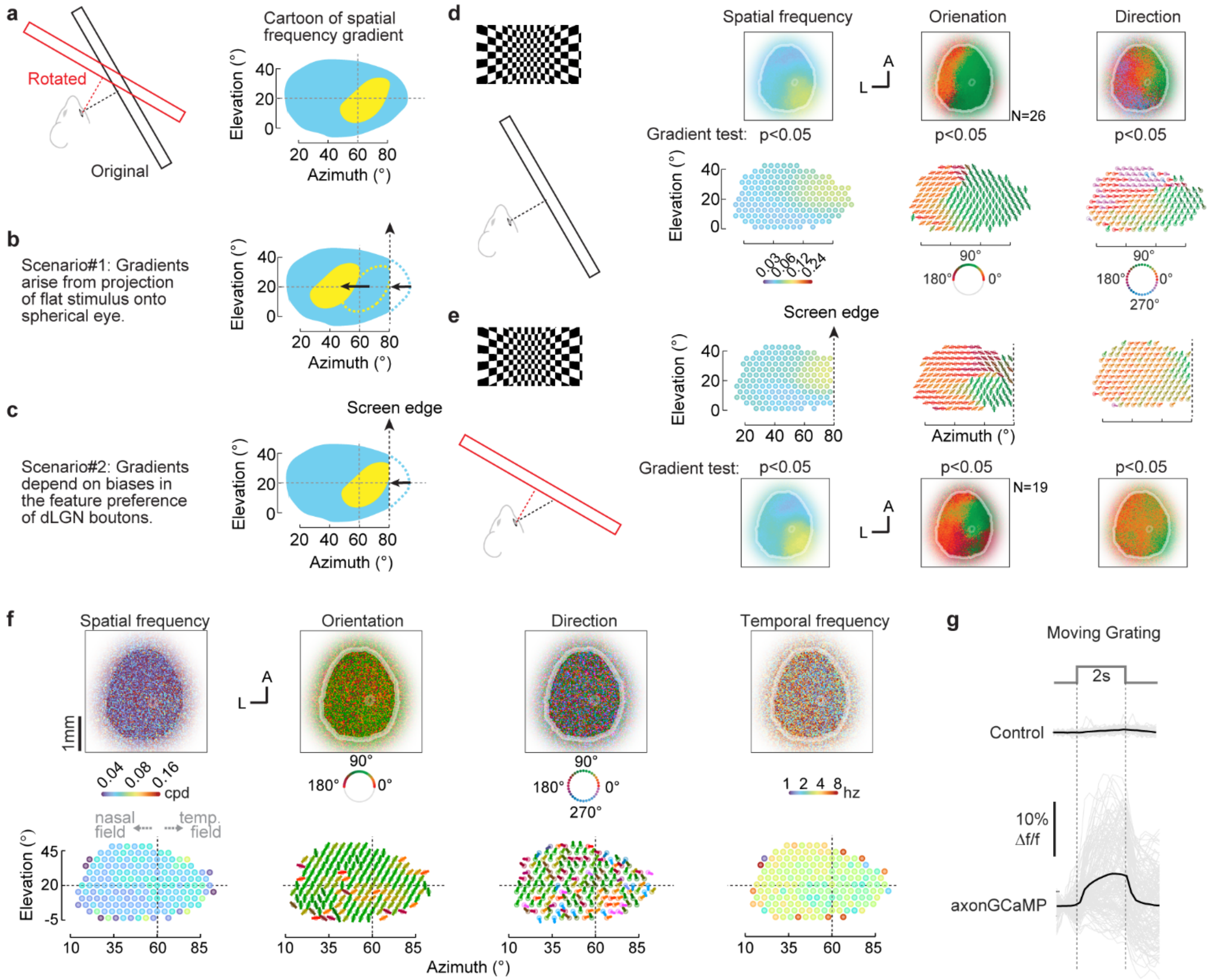

Figure S4. dLGN feature gradients are unaffected by fisheye distortion and screen rotation and are not the result of autofluorescence. a. Left: Cartoon illustrates a mouse's head relative to our original monitor position (black) and the rotated position (red). Dotted lines are perpendicular to the screen surface go from screen center to optical center of the mouse eye. Right: Schematic of spatial frequency gradient obtained with non-rotated screen. **b.** Scenario#1: spatial frequency gradient center should rotate nasally with the rotated screen if it is produced by the projection of a flat stimulus onto the spherical eye. **c.** Scenario#2: spatial frequency gradient center should be unaffected by screen rotation if feature biases have biological origin. **d.** Top: Average preferred feature maps of dLGN boutons across V1 with our original screen position. Bottom: Maps from top transformed into 2.5º hexagonal bins across visual space. The mouse optical axis (60,20). Anterior (A) and lateral (L) indicated. **e.** Same as d, but with rotated screen configuration. Map structure does not appreciably rotate with the screen indicating that gradients arise from biases in the feature preference of dLGN boutons. Note that seven animals underwent multiple sessions of experiments for both conditions (n = 26 sessions for d; and n = 19 session for e). **f.** *top:* Average preferred feature maps computed from widefield imaging a control animal that did not express axonGCaMP8s. *bottom:* Maps from Panel a transformed into 2.5º hexagonal bins across visual space. **g.** Average Δf/f aligned to the onset of a 2 second moving grating stimulus from the same animal as shown in f (control). Average from a mouse that express axon-localized GCaMP8s are shown for comparison. Gray lines are individual repetitions. Anterior (A) and lateral (L) indicated.

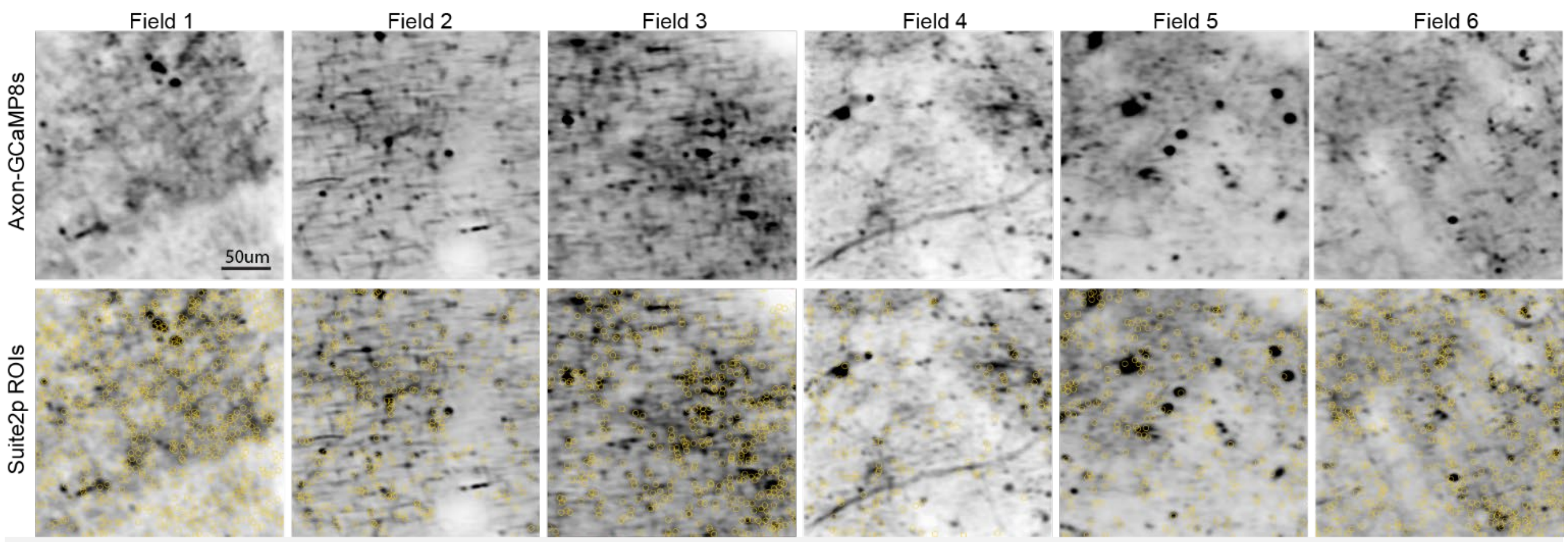

Figure S5. Example 2p fields of axon-GCaMP8s boutons. Example two-photon fields containing axon-GCaMP8s expressing boutons (top) and the same fields overlayed with ROIs showing statistically significant responses among those defined by Suite2p (yellow circles, bottom). Scale bar indicated.

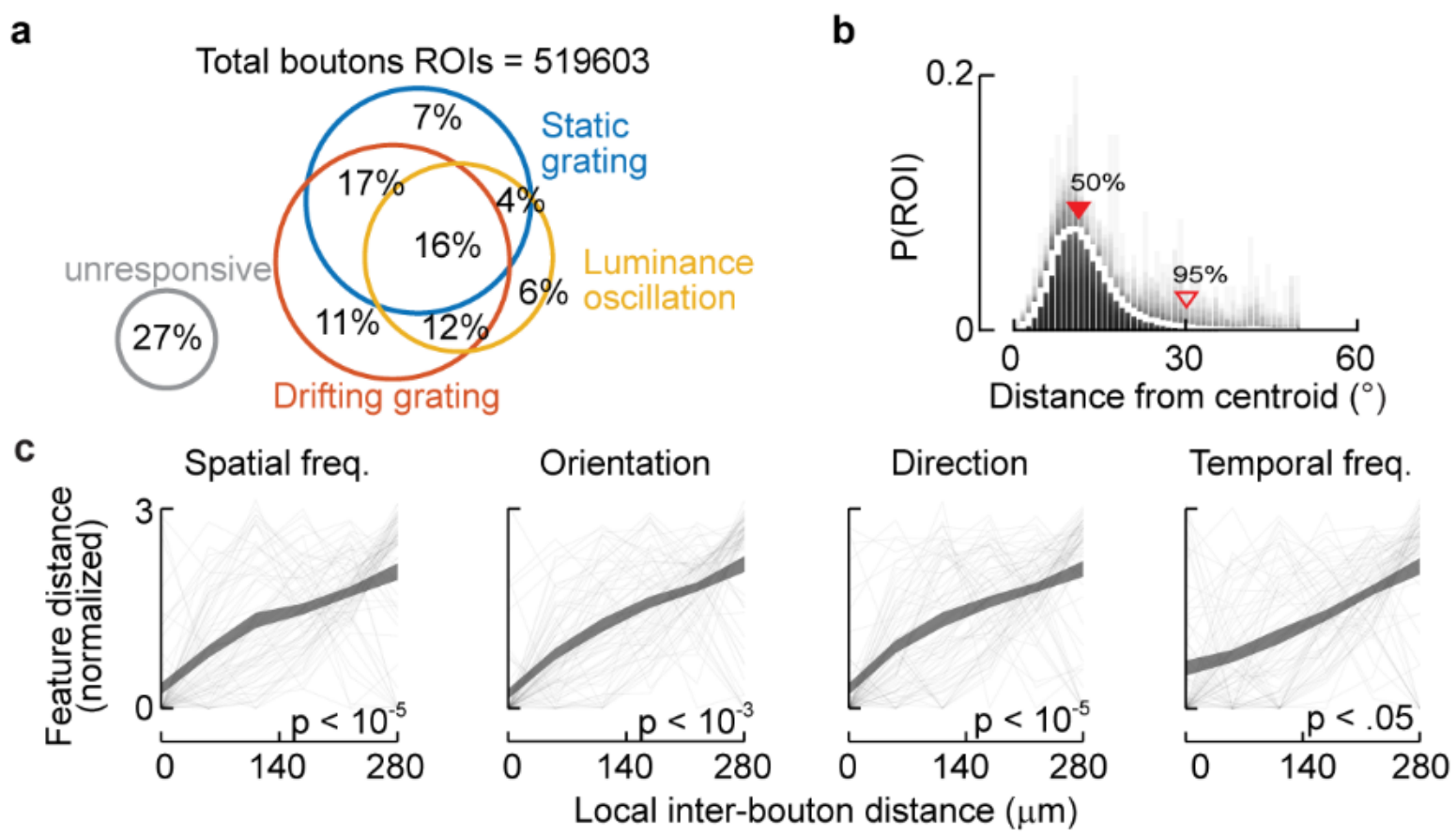

Figure S6. Bouton selection, similarity within and across two-photon fields. a. Proportions of responsive boutons out of 519,603 regions of interest (ROI) identified by Suite2p. Responsiveness was tested with t-tests that compared the response of each ROI to their preferred stimulus within each stimulus set. Each of the 3 ROI sets was used for the respective feature analysis. **b.** Scatter of receptive field (RF) within the field measured by deviation (distance) from the population RF center of the field. Only responsive ROIs (379,311) were included. The population RF center was computed as the median location of ROIs within the field. Histograms of each field are overlaid in gray and the white contour represents the average distribution across all fields. Triangles indicate 50- and 95-percentiles. Most boutons (95%) fall within ~30 degrees of their population RF centroid. ROIs with RFs greater than 30 degrees were excluded from analysis. **c.** Monotonic increase in feature distance as a function of inter-bouton distance within the field. Data from 67 fields are plotted as thin lines overlaid by a thick line for mean ± SEM. Slopes of linear fit to individual field data were significantly above zero ($p < 0.05$ in all 4 feature domains, t-test, n = 67 fields, 11 mice).

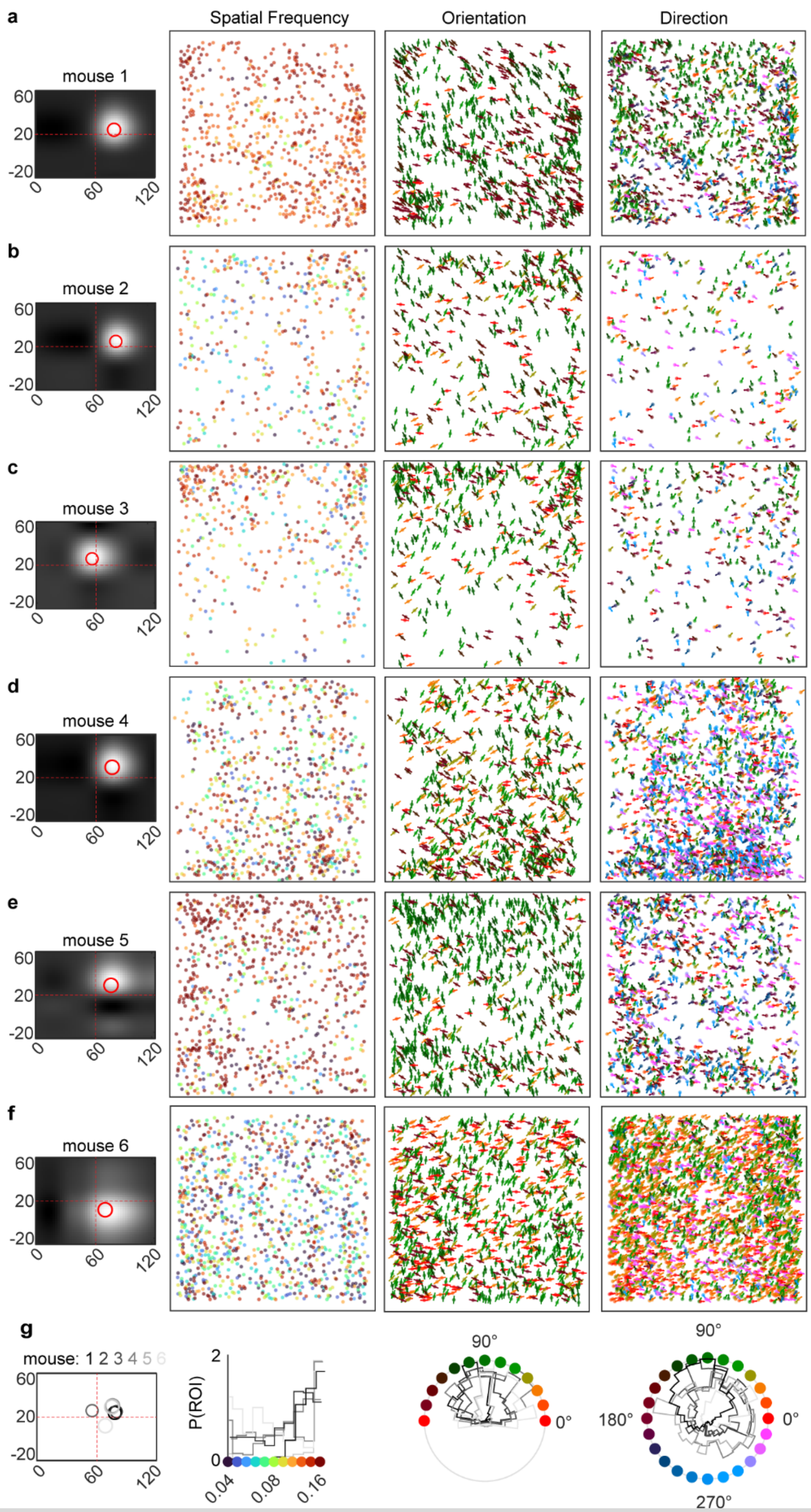

Figure S7. Visual field matched examples from different mice. a-f. Example 2p fields from 6 individual mice. From left to right: population receptive field estimate, boutons responsive to spatial frequency, orientation, and directional motion. **g.** Summary graphs compare the 6 mice shown in a-f. Screen center and optical axis is (60,20). Visual comparison within a given feature (i.e., spatial frequency, orientation, direction) shows qualitative similarity. Fields of spatial frequency preferring boutons tend to be generally "red" (high spatial frequency); fields of orientation preferring boutons tend to be generally "green" (vertical bias); and fields of direction preferring boutons tend to span a range from pink/red to green (posterior/upward motion). These patterns match average preferences in Figure 2g.

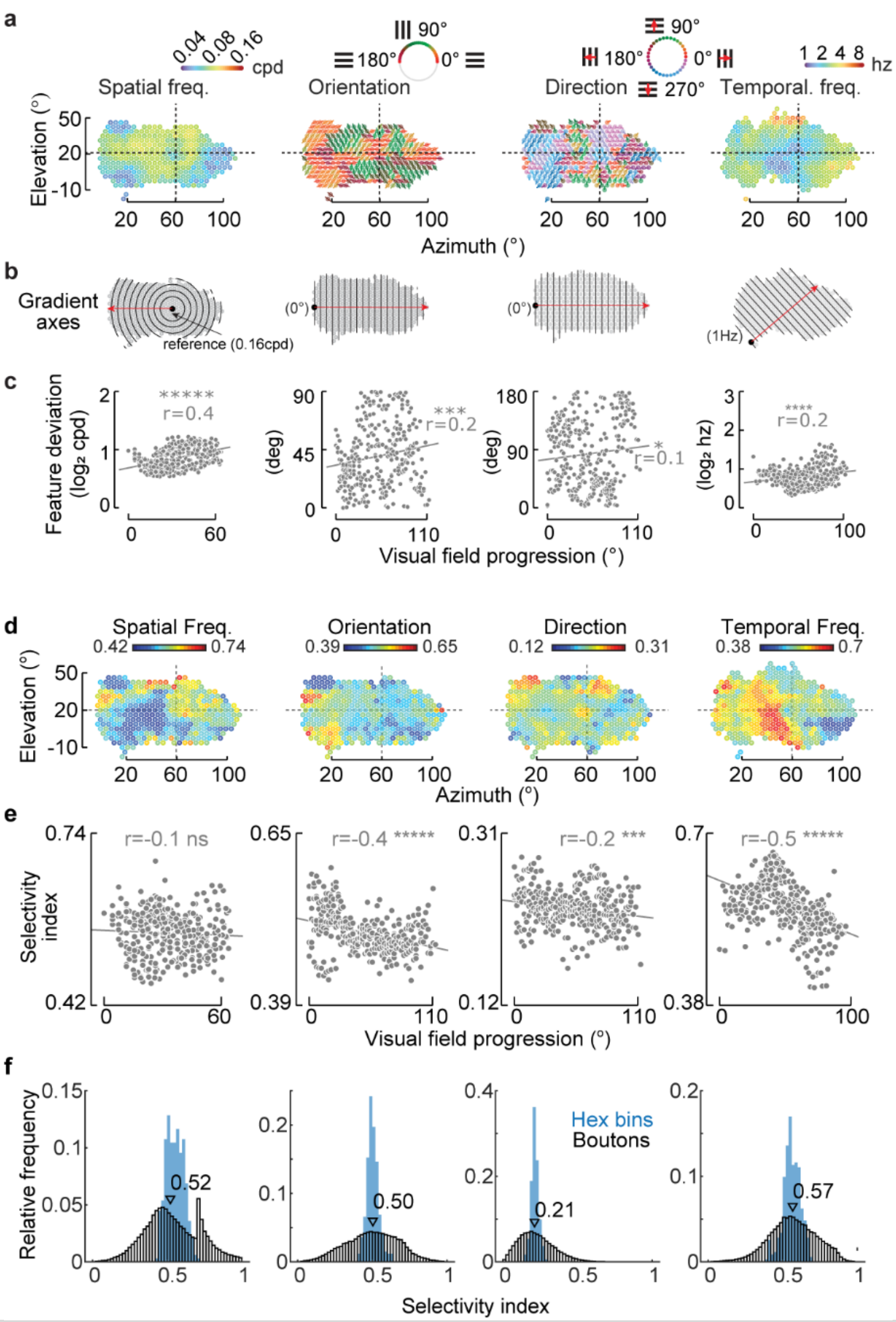

Figure S8. 2p bouton feature maps and gradients after neuropil signal correction by Suite2p and feature selectivity across visual locations. a. Spatial distribution of preferred features in the same format as Fig 2g from a neuropil-corrected dataset. **b.** Gradient axes for c. The same as Fig. 2h. **c.** Feature gradients in the same format as Fig 2i.(r: Pearson's correlation, one-sided test, ***** $p < 10^{-10}$; **** $p < 10^{-5}$; *** $p < 10^{-3}$; and * $p < 0.05$). Significantly positive r-values indicate that preferences change linearly along the same feature axis as in widefield imaging). **d.** Bouton feature selectivity from 2p fields across layers binned using 2.5 deg hexagonal bins across the visual field. **e.** Selectivity index of each bin versus visual field progression along characteristic axes (n = 332 [SF/Ori], 347 [Dir], and 344 [TF]; 11 mice and 67 fields). **f.** Histograms of feature selectivity index for hexagonally binned bouton data (blue) or raw boutons (black). Open triangles indicate mean of raw bouton distributions.

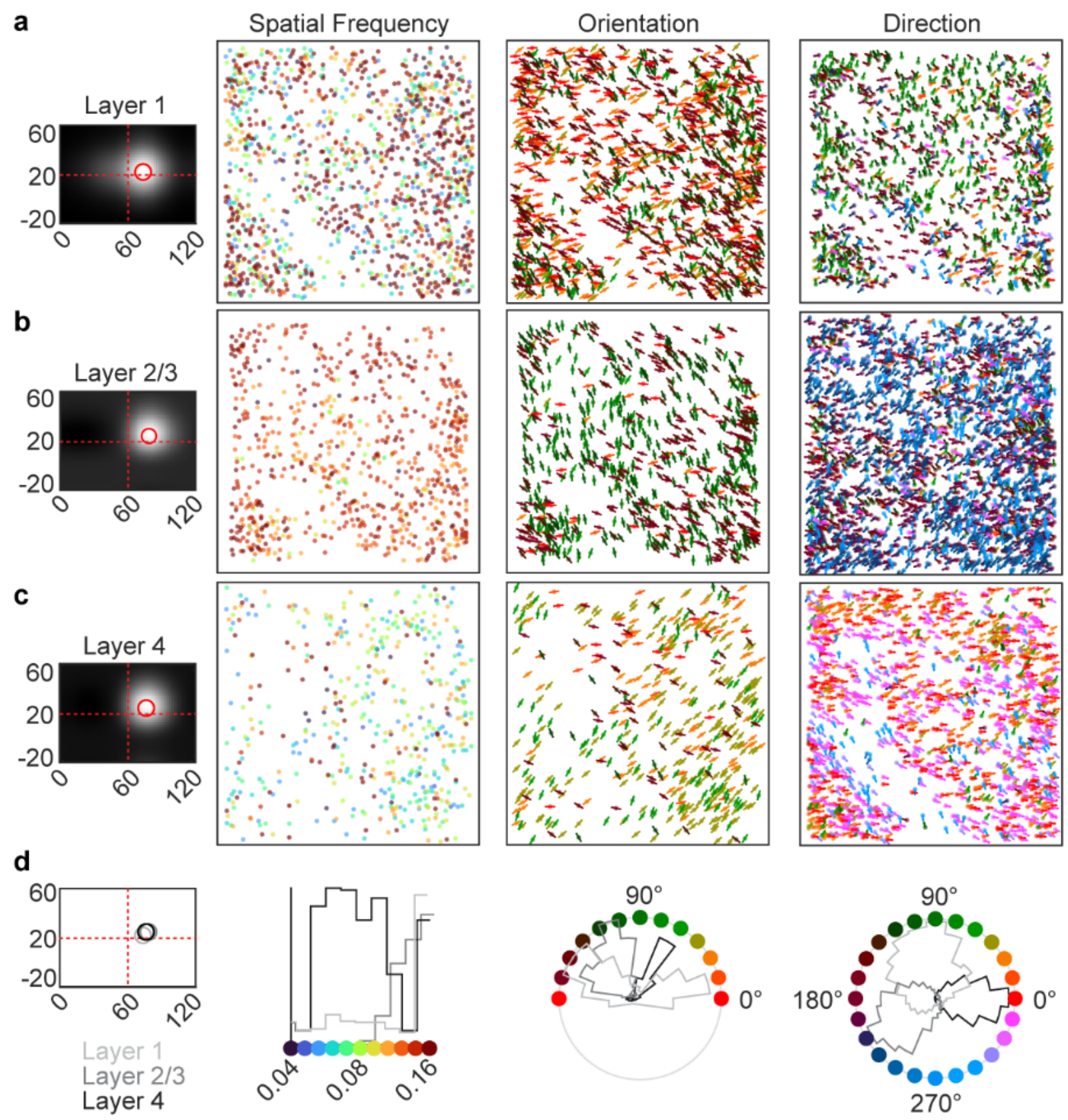

Figure S9. Receptive field matched examples from different cortical layers. a-c. Example 2p fields from layer 1 (a), layer 2/3 (b), and layer 4 (c). From left to right: population receptive field estimate, boutons responsive to spatial frequency, orientation, and directional motion. **d.** Summary graphs compare boutons in a-c. Screen center and optical axis is (60,20). Visual comparison within a given feature showed qualitative similarities. Fields of spatial frequency preferring boutons tended to be generally 'yellow-red' (high spatial frequency). Fields of orientation preferring boutons were 'green' in layers 2/3 and 4 (vertical orientation) but were more 'red' in layer 1, consistent with our orientation gradients in Figure 3c. Fields of direction preferring boutons spanned a range from green to pink in layers 1 and layer 4 (posterior motion), but were more 'blue-brown' in layer 2/3, consistent with the direction gradients in Figure 3c.

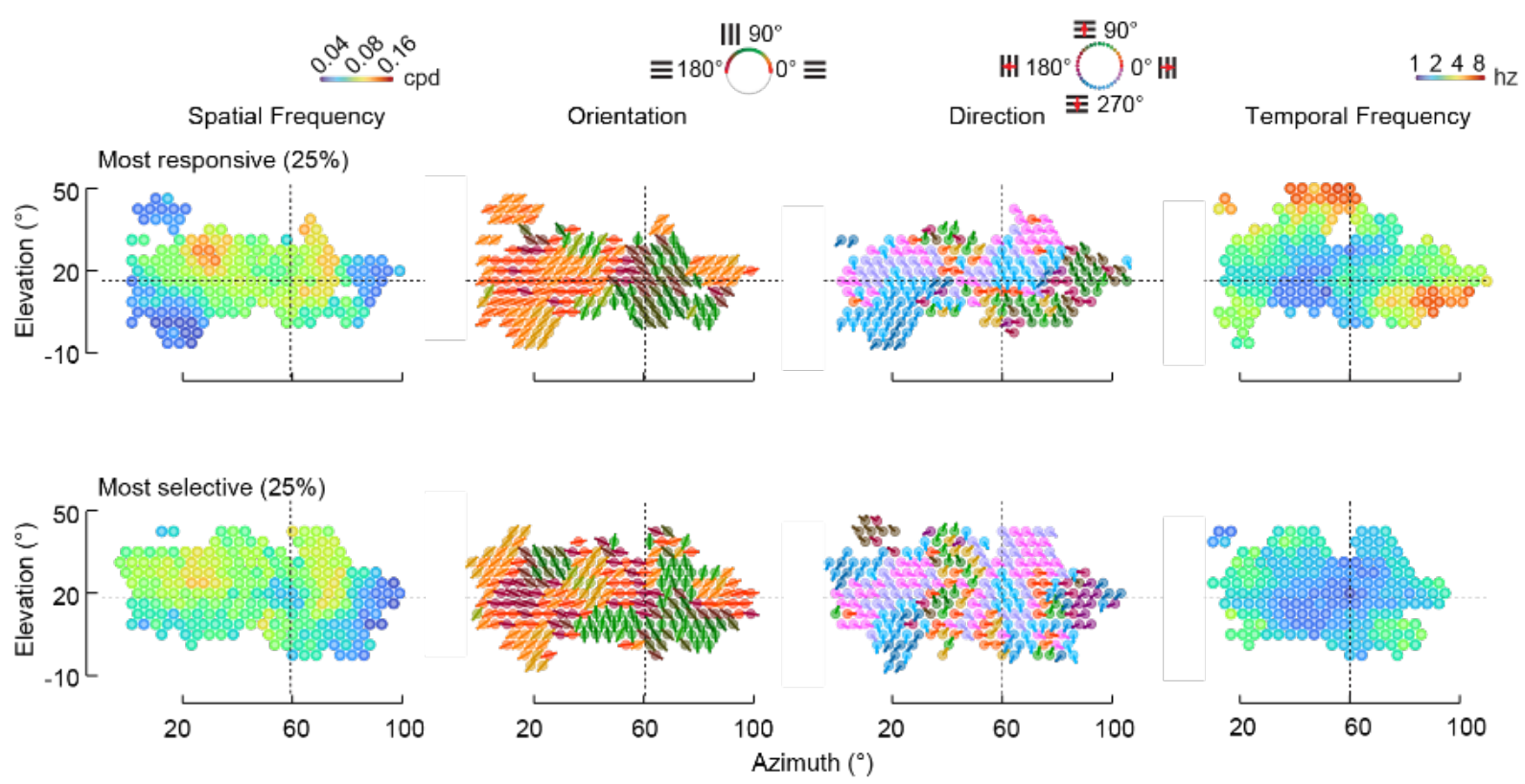

Figure S10. 2p bouton feature maps of the most responsive vs the most selective ROIs. Bouton feature preference maps for spatial frequency, orientation, direction, and temporal frequency binned across the visual field out of top 25% responsive ROIs (top row) and top 25% selective ROIs (bottom row) Individual bouton data were re-binned in each panel and bins with less than 30 boutons were discarded, which yielded different numbers of bins in each panel (n>=127 per bin).

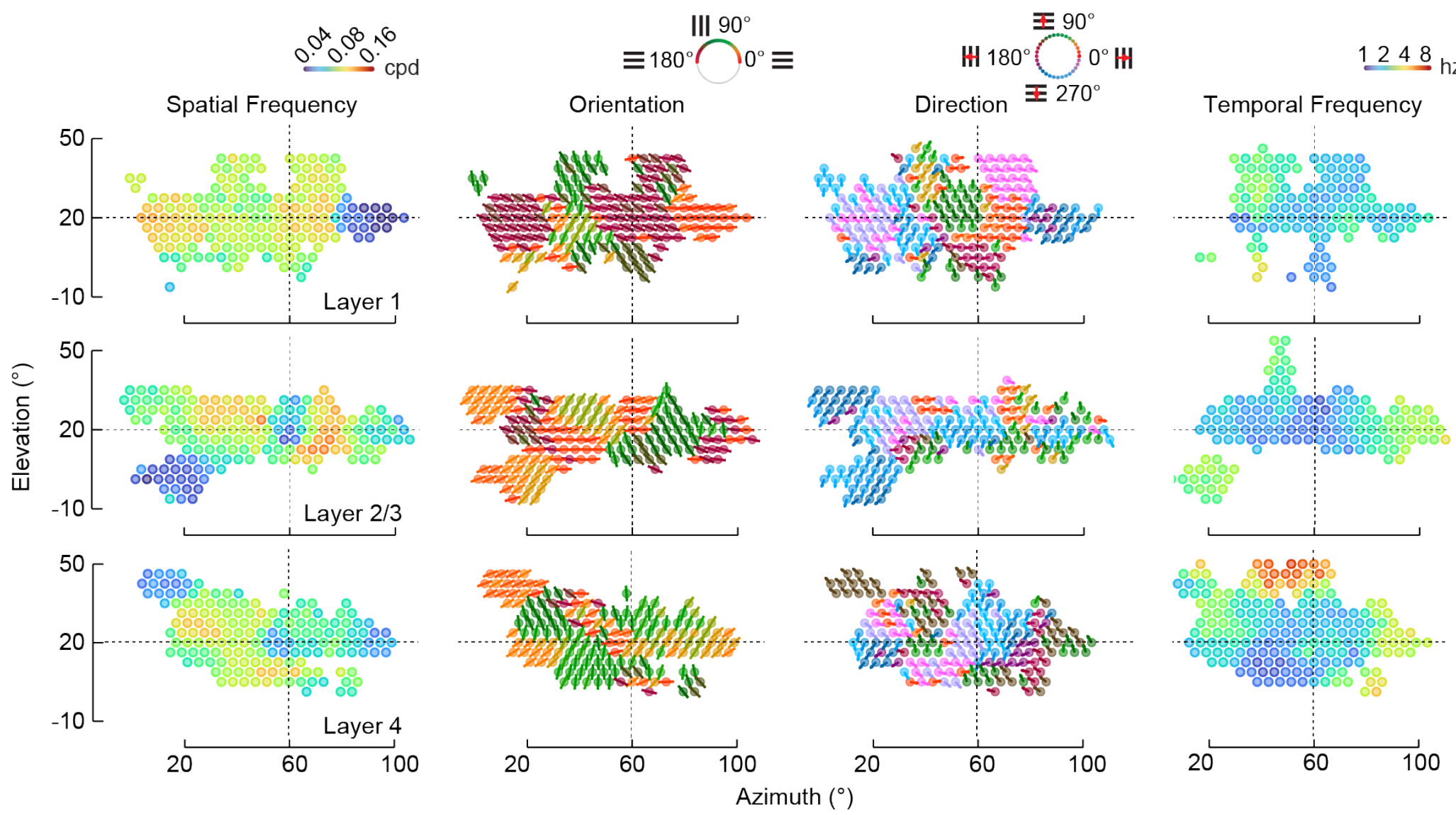

Figure S11. 2p bouton feature maps across cortical layers. Bouton feature preference maps for spatial frequency, orientation, direction, and temporal frequency binned across the visual field from layer 1 (top row), layer 2/3 (middle row), and layer 4 (bottom row). Out of 67 fields, we chose 48 which had the whole set of 3 layers along the same retinotopic location (16 locations x 3 depths). Individual bouton data were re-binned in each panel and bins with less than 30 boutons were discarded, which yielded different numbers of bins in each panel (n>=127 per bin).

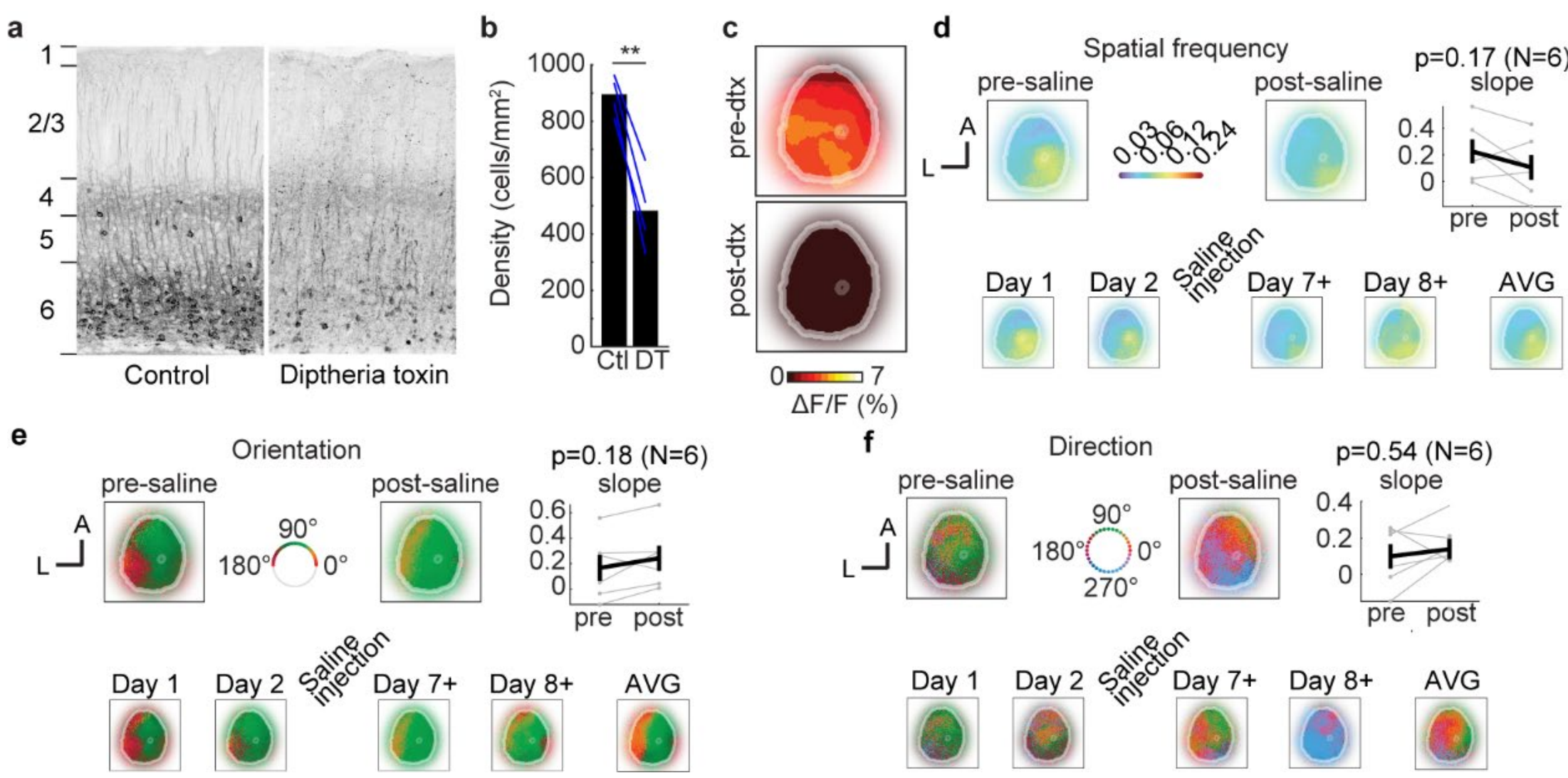

Figure S12. Controls for DTR ablation experiments. a. Cortical sections showing Reporter+/DTR+ layer 6 neurons following injection with saline or DT. **b.** Quantification of cell counts of experiments like those shown in b. ** is p < 0.01 t-test. **c.** Average population Δf/f of GCaMP6f+, diphtheria toxin receptor-positive layer 6 cortical neurons to a drifting grating (top) and the same average 7 days after injection of diphtheria toxin (dtx, bottom). DTX produces uniform loss of layer 6 activity (n=3). **d-f.** Pre-saline and post-saline average maps across 6 mice (1-2 sessions each) for preferred spatial frequency (d), orientation (e), and direction (f). Slopes of the gradient model are plotted to the right in each panel (dots: individual mice; and thick black lines: mean ± std). Bottom row shows average across mice for corresponding sessions There was at least 5 days between the second session (Day 2) before saline injection and the first session after injection (Day 6+). Anterior (A) and lateral (L) indicated.

Table S1: Mean, standard error, effect size, and sample sizes for Figures 1-5.

Spatial frequency								
**Dataset**	**Fig number**	**Slope (Octaves per 100 degrees)**		**Effect size***		**P-value**	**Sample size**	**Number of animals/retinas**
		Mean	SE	Cohen's d	Correlation (adjusted r^2)			
Wide field	1	1.72	0.26	1.12	N/A	4.46E-08	36	36
Wide field - Control	5	1.76	0.42	0.96	N/A	3.88E-04	18	18
Wide field - L6 ablation	5	1.04	0.24	1.03	N/A	2.10E-04	18	18
Wide field - Change	5	-0.67	0.38	-0.41	N/A	4.86E-02	18	18
Two-photon	2	0.69	0.08	N/A	0.42 (0.18)	4.95E-16	332	11
- Most responsive	3	1.27	0.17	N/A	0.59	2.11E-19	188	11
Retina	4	0.29	0.08	N/A	0.16 (0.03)	2.77E-04	428	9

Orientation								
**Dataset**	**Fig number**	**Slope (Degrees per 100 degrees)**		**Effect size***		**P-value**	**Sample size**	**Number of animals/retinas**
		Mean	SE	Cohen's d	Correlation (adjusted r^2)			
Wide field	1	20.54	5.84	0.59	N/A	4.46E-08	36	36
Wide field - Control	5	12.04	6.71	0.42	N/A	3.88E-04	18	18
Wide field - L6 ablation	5	7.13	5.02	0.36	N/A	2.10E-04	18	18
Wide field - Change	5	-4.91	7.87	-0.15	N/A	2.71E-01	18	18
Two-photon	2	14.82	4.5	N/A	0.18 (0.03)	5.54E-04	332	11
- Most responsive	3	22.34	6.24	N/A	0.24 (0.05)	2.17E-04	219	11
Retina	4	11.6	3.9	N/A	0.14 (0.02)	1.62E-03	428	9

Direction

Dataset	Fig number	Slope (Degrees per 100 degrees)		Effect size*		P-value	Sample size	Number of animals/retinas
		Mean	SE	Cohen's d	Correlation (adjusted r^2)			
Wide field	1	15.76	9.01	0.29	N/A	4.46E-02	36	36
Wide field - Control	5	32.43	11.27	0.68	N/A	5.21E-03	18	18
Wide field - L6 ablation	5	22.1	12.69	0.41	N/A	4.98E-02	18	18
Wide field - Change	5	-10.33	18.79	-0.13	N/A	2.95E-01	18	18
Two-photon	2	20.594	9.16	N/A	0.12 (0.01)	1.27E-02	347	11
- Most responsive	3	34.77	11.45	N/A	0.21 (0.04)	2.17E-04	201	11
Retina	4	-2.64	7.26	N/A	-0.02 (0.00)	6.42E-01	428	9

Temporal frequency

Dataset	Fig number	Slope (Octaves per 100 degrees)		Effect size*		P-value	Sample size	Number of animals/retinas
		Mean	SE	Cohen's d	Correlation (adjusted r^2)			
Wide field	1	1.34	0.23	0.99	N/A	4.94E-07	36	36
Wide field - Control	5	1.38	0.35	0.94	N/A	4.74E-04	18	18
Wide field - L6 ablation	5	0.63	0.32	0.46	N/A	3.37E-02	18	18
Wide field - Change	5	-0.75	0.35	-0.5	N/A	2.37E-02	18	18
Two-photon	2	0.39	0.08	N/A	0.25 (0.06)	1.31E-06	344	11
- Most responsive	3	1.1	0.18	N/A	0.38 (0.14)	5.01E-09	208	11
Retina	4	0	0.04	N/A	-0.00 (0.00)	5.17E-01	407	9

* = Effect size is listed as either Cohen's d or Correlation (adjusted r^2)
